# Quantifying Overlapping Forms of Malnutrition Across Latin America: A Systematic Literature Review and Meta-Analysis of Prevalence Estimates

**DOI:** 10.1016/j.advnut.2024.100212

**Published:** 2024-03-15

**Authors:** Diana Sagastume, Antonio Barrenechea-Pulache, Andrea Ruiz-Alejos, Katja Polman, Lenka Beňová, Manuel Ramírez-Zea, José L Peñalvo

**Affiliations:** 1 Department of Public Health, Institute of Tropical Medicine, Antwerp, Belgium; 2 Global Health Institute, University of Antwerp, Wilrijk, Belgium; 3 Facultad de Medicina, Universidad Científica del Sur, Miraflores, Lima, Perú; 4 Department of Health Sciences, Vrije Universiteit (VU) Amsterdam, Netherlands; 5 INCAP Research Center for the Prevention of Chronic Diseases (CIIPEC), Institute of Nutrition of Central America and Panama (INCAP), Guatemala City, Guatemala; 6 National Center for Epidemiology, Carlos III Institute of Health (ISCIII), Madrid, Spain

**Keywords:** malnutrition, double burden of malnutrition, Latin America, the Caribbean, systematic review, meta-analysis, stunting, obesity

## Abstract

Estimating the prevalence of double burden of malnutrition (DBM) is challenging in the Latin American and Caribbean (LAC) region where various DBM typologies (e.g., obesity and stunting) are heterogeneous and estimates are scattered across literature This study aimed to assess the prevalence of DBM typologies in the LAC region. We searched PubMed, Embase, Scopus, and Web of Science to identify studies on the prevalence of DBM published between 1 January, 2000, and 23 January, 2023. Outcomes were the prevalence of the identified DBM typologies at the household, individual, or across life course levels. Random-effect meta-analyses of proportions were used to estimate pooled period prevalence for all outcomes. Heterogeneity was explored using meta-regressions. From 754 records identified, 60 (8%) studies were eligible, with a median of 4379 individuals. Studies reported data from 27 LAC countries collected between 1988 and 2017. Most studies used nationally representative surveys (68%) and scored as low risk of bias (70%). We identified 17 DBM typologies for which 360 estimates were analyzed. The prevalence of the identified DBM typologies ranged between 0% and 24%, with the DBM typology of “adult with overweight and child with anemia” having the highest prevalence (24.3%; 95% CI: 18.8%, 30.2%). The most frequently reported DBM typology was “adult with overweight and child with stunting,” with a prevalence of 8.5% (95% CI: 7.7, 9.3). All prevalences carried large heterogeneity (*I*^2^>90%), modestly explained by subregions and countries. DBM across the life course could not be estimated owing to insufficient estimates. In conclusion, using available data, our study suggests that the burden of DBM in the LAC region ranges between 0% and 24%. In the most frequent DBM typologies, overweight was a common contributor. Substantial progress can be made in curbing the burden of DBM in the LAC region through strategies addressing excess weight within these population groups. This study was registered at PROSPERO as CRD42023406755.

**Statements of significance**

This study’s findings suggest that the prevalence of double burden of malnutrition (DBM) ranges from 0% to 24% in the Latin America and Caribbean (LAC) region, hence underscoring the urgency of implementing evidence-based strategies to curb DBM and, particularly, to address overweight in the region. To our knowledge, this study is the first attempt to map out and measure the DBM phenomena in the LAC region, highlighting both the existing data and evidence gaps. We intend for this study to become a source of information and reference for international health agencies, countries’ governments, and funding organizations to provide insight into the magnitude and heterogeneity of DBM as a public health issue in the LAC region, guide future research, and support decision-making and investments to improve the health and development of this region.

## Introduction

In line with the sustainable development goals (SDG), particularly SDG2, the United Nations Decade of Action on Nutrition 2016–2025 is committed to a decade-long concerted effort to implement policies, programs, and enhanced investments to eradicate all forms of malnutrition [1,2]. Malnutrition, including under and overnutrition, remains a global health issue and is considered a silent pandemic [3], contributing notably to the global disease burden [4]. In 2019, it was estimated that 50 million Disability-Adjusted Life Years (DALYs) globally were related to undernutrition and 160 million DALYs to obesity [5]. In the World Bank region classification of Latin America and the Caribbean (LAC) region [6], where 59% of the 42 countries are classified as low-income and middle-income countries [7], the rates of undernutrition are gradually decreasing, whereas obesity has risen steeply over the last years [8,9]. For children younger than 5 years, between 2012 and 2022, a decrease in stunting from 12.7% to 11.5% and wasting from 1.4% to 1.3% and an increase in overweight from 7.4% to 8.6% has been observed [10]. On the contrary, between 2000 and 2016, among adults aged 18 years and older, overweight and obesity rates increased from 49.6% to 59.5% and 16.6% to 24.2%, respectively [9]. Similarly, in 5 years, 2012–2016, anemia has increased from 21.2% to 22.0% among women of reproductive age [9].

Many low-income and middle-income countries are confronted with the coexistence of both burdens, undernutrition and overnutrition [11], which are characteristics of intermediate stages of the demographic, epidemiologic, and nutrition transitions [12]. This situation is referred to as the double burden of malnutrition (DBM), which is defined by the WHO as “the coexistence of undernutrition along with overweight, obesity or diet-related noncommunicable diseases (NCDs), within individuals, households, populations, and across the life course” [13]. This definition is highly conceptual, making its application challenging and complex to characterize the epidemiology of DBM, because it covers multiple indicators and combinations across individual, geographical, and temporal levels. Furthermore, it assigns the same public health relevance and probability of occurrence to the different levels of DBM. Although this is effective in conveying the overall message and importance, the nuances of this definition and its applicability in real settings remain unknown. Moreover, the magnitude of DBM, including all possible DBM typologies (combinations of indicators at different levels), has not been estimated in the LAC region. Studies often focus only on assessing DBM at a national level [14], on evaluating only 1 DBM typology (e.g., a mother with overweight and a child with stunting) [15] or on a specific population subgroup (children younger than 5 years) [16]. Moreover, research is often based on the same data sources, primarily the Demographic and Health Survey (DHS) [17,18]. In addition, other forms of undernutrition, such as micronutrient deficiencies, are frequently overlooked in the available literature, leading to potential underestimation of DBM [19].

This study aimed to quantitatively assess the period prevalence of DBM typologies within the LAC region, based on a rigorous systematic review of existing evidence, to provide a thorough overview of the DBM’s situation over the past 2 decades and identify research gaps to guide future research. By doing so, we intend to estimate the magnitude of the burden of DBM on the public health system of this region and advocate for immediate and concerted efforts to address it.

## Methods

This study was conducted following the Preferred Reporting Items for Systematic Reviews and Meta-analysis (PRISMA) guidelines [20] and registered in the International Prospective Register of Systematic Reviews (PROSPERO; CRD42023406755). Deviations from the original protocol are presented in Supplemental Section 1.1.

### Definition

**FIGURE 1:**
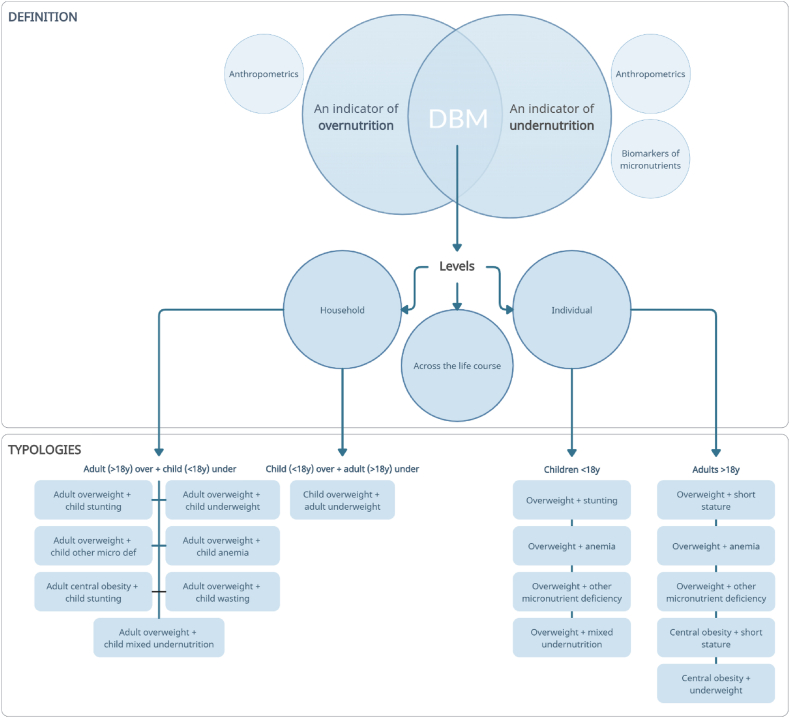
DBM definition and DBM typologies identified in the eligible evidence. For children, anthropometrics included overweight (weight-for-height or BMI-for-age), stunting (height-for-age), wasting (weight-for-age or weight-for-height), underweight (weight-for-age or BMI-for-age), and mixed undernutrition (combination of the aforementioned methods). For adults, anthropometrics included overweight (BMI), central obesity (waist circumference, waist-to-hip ratio, and waist-to-height ratio), short stature (height cutoff specific by each study), and underweight (BMI). Biomarkers of micronutrients included, for both children and adults, anemia (laboratory cutoff by each study) and other micronutrient deficiencies (laboratory cutoff by each study). To optimize the analyses the following classification was made: child/children refers to those aged younger than 18 years and adults to those aged 18 years and older; over refers to types of overnutrition and under to types of undernutrition. DBM, double burden of malnutrition.

In this study, DBM was defined as the coexistence of an indicator of undernutrition and an indicator of overnutrition at the levels of the household (including mother–child pairs), individuals, or across the life course (Figure 1).

### Search strategy and selection criteria

Preliminary searches of relevant references and reviews were conducted in PubMed-MEDLINE and PROSPERO to identify the available literature and research gaps and conceptualize the study. A systematic search was carried out on PubMed-MEDLINE, Embase, Scopus, and Web of Science to identify studies that evaluated the prevalence of DBM in any LAC country [6], published in English, French, Portuguese, or Spanish between 1 January, 2000, and 23 January, 2023, date when the search was conducted. The search strategy included a combination of concepts, keywords, and synonyms for DBM and the list of countries in the LAC region (Supplemental Section 1.2). Intending to capture all available evidence, our search was complemented by manual searches of references of the retrieved reviews.

Studies were eligible if they had an observational design (including cross-sectional, cohorts, or case–control) and were conducted in any country of the LAC region according to the World Bank regions classification [6]. Eligible studies had to relate to populations of participants of any age and sex but without evidence of any severe chronic condition (e.g., cancer) to avoid malnutrition outcomes as a consequence of these conditions. The studies needed to include ≥2 coinciding malnutrition outcomes, one assessing undernutrition and the other overnutrition (e.g., stunting and obesity), measured at the levels of the household, individual, or across the life course. The malnutrition outcomes needed to be measured using objective instruments. Studies must provide a prevalence estimate of DBM reported in percentages or as the frequency of cases in the total study population. The exclusion criteria are presented in Supplemental Section 1.3. Two investigators (DS and AB-P) performed the title and abstract screening and full-text assessment of potentially eligible studies in duplicate and independently. Discrepancies were resolved by discussions and consensus.

### Data extraction

Data were extracted using the online tool Covidence [21] and complemented with a standardized electronic Excel template developed by the investigators. In Covidence, information was extracted about each study including the author(s), year of publication, study design, setting details, and population characteristics. On the Excel template, information pertinent to the DBM estimate was extracted comprising data collection details, country, year of data collection, and DBM specifications, particularly the level of assessment, definition, typology, indicator, and prevalence estimate as the number of cases and total study population (Supplemental Section 1.4). To optimize the analyses and the text in the manuscript, child/children refers to those aged younger than 18 years and adults to those aged 18 years and older.

### Quality assessment

Owing to the different study designs of the eligible evidence, 2 tools were applied to assess the methodological quality of the studies. For cross-sectional designs, the tool developed by Hoy et al. [22] was used, to assess the risk of study bias by assigning a score of 1 (if present) or 0 (if not present) to 10 items measuring the internal and external validities of the study. A final score was calculated by summing up the individual scores, ranging between 0 and 10, and classified as evidence of low (>8), moderate (6–8), and high (≤5) risk of bias. For the few cohort studies, a tool developed by the NIH’s National Heart, Lung, and Blood Institute (NHLBI) was used [23]. This study summed a score ranging between 0 and 14, which classified the studies as having low (≥12), moderate (8–11), and high (≤7) risk of bias.

Data extraction and quality assessment of the studies were conducted independently by 2 investigators (DS and AB-P or AR-A or JLP). Differences were resolved by discussions and the involvement of a third investigator when necessary. If unclear or absent information, study authors were contacted via email; if no response was obtained, conservative assumptions were made when feasible (Supplemental Table 1).

### Outcomes

Based on our DBM definition, DBM typologies were identified in the eligible studies. Our outcomes entailed the 17 identified DBM typologies, which are presented in Figure 1 and Supplemental Table 2. At the individual level, DBM typologies were differentiated for children and adults. As most studies reported >1 DBM typology, each typology was considered independent and included as a separate estimate in the analyses. Similarly, because the majority of studies selected a study population based on specific eligibility criteria, if multiple studies used the same data source and estimated the same malnutrition outcome, all estimates were retained and analyzed as independent estimates. Owing to the small number of DBM typology estimates that included micronutrient deficiencies (e.g., vitamin A deficiency or zinc deficiency) other than anemia as the type of undernutrition, these estimates were combined into a category of undernutrition named “other micronutrient deficiencies.” Similarly, estimates that included mixed types of undernutrition (stunting and anemia) were also combined into a category of undernutrition called “mixed undernutrition.”

### Data analysis

For the systematic review of the eligible studies, information was compiled in evidence tables to facilitate description and comparisons. Study characteristics (design, setting, and population) and DBM specifications (data collection information, types of malnutrition, and DBM level and definition), were quantified using measures of central tendency for continuous variables and frequencies and percentages for categorical variables. DBM specifications were summarized based on the total number of estimates extracted. For the systematic review and descriptive statistics, all eligible studies and DBM estimates were included independently of being or not included in the meta-analysis, as is the case of studies and estimates measuring DBM across the life course.

To ease the reading of the article, from this point onward the term prevalence will be used to refer to “pooled period prevalence.” To quantify the pooled prevalence and 95% CI, of each DBM typology, a random-effect meta-analysis of proportions (command *metaprop* in Stata) was used. For this, the study-specific estimates of each DBM typology, which were extracted from the eligible studies, were analyzed using 2 necessary inputs: *1*) the absolute number of cases and *2*) the total number of population. Freeman–Tukey double arcsine transformation was used to stabilize variances from extreme proportional estimates. Meta-analyses were conducted for the outcomes with a minimum of 4 DBM estimates. Between-study heterogeneity was assessed by Cochran Q and *I*^2^ statistics. If substantial levels of heterogeneity (*I*^2^ > 75%) were present in the prevalence of DBM typologies, univariate and multivariable meta-regressions were conducted to identify prespecified sources of heterogeneity. The meta-regressions consisted of the categorical variables of LAC subregion, country, country income, area type, year of data collection, number of participants (based on the median for each outcome), and risk of bias. Meta-regressions were performed only for those DBM typologies with >10 estimates. Stratified meta-analyses were conducted based on significant sources of heterogeneity singled out by meta-regressions. To explore whether there is an indicator of the evolution over time of DBM, we carried out stratified meta-analyses based on categories of the year of data collection (1988–2000, 2001–2011, 2012–2017) for the 2 outcomes with the largest number of estimates. Sensitivity analyses were also carried out to evaluate whether non-nationally representative studies influenced the results. For all analyses, a *P* < 0.05 was considered statistically significant, except for the multivariate meta-regressions where a Bonferroni-adjusted *P* value of <0.007 was considered owing to multiple comparisons. Analyses were conducted in Stata (Release 16/SE; StataCorp LP). A narrative description of the studies measuring DBM across the life course was provided.

## Results

**FIGURE 2:**
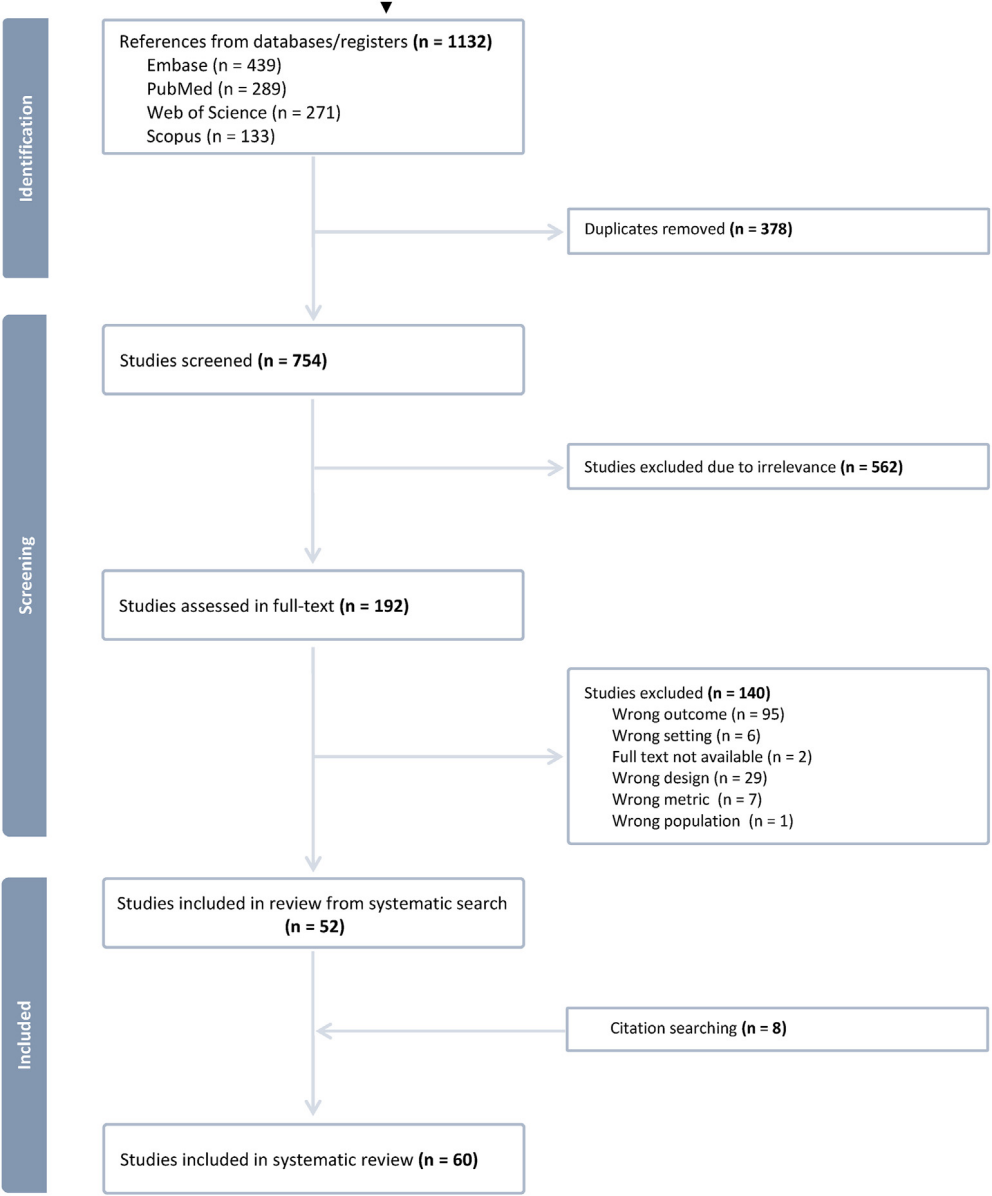
Flowchart—study selection process of eligible studies.

Initially, our search identified 754 unique references of which 52 studies were eligible. Eight additional studies were identified through the manual searches of references of retrieved reviews (Figure 2). A total of 60 studies were included and are described in Supplemental Table 3. The main characteristics of the eligible studies are presented in Table 1. Half of the studies (52%) were published between 2016 and 2023; most had a cross-sectional design (95%), used a nationally representative survey (68%), mainly DHS (25%), the combination of DHS and other data sources (10%), or other National Nutritional Surveys (20%). Nearly, a quarter (23%) used other non-nationally representative data sources (e.g., the Global School-Based Student Health Survey), and some others were unspecified (9%). Most studies (70%) were classified as low evidence of risk of bias and 30% as moderate risk. Geographically, most studies were conducted in South America (53%) [18,[24], [25], [26], [27], [28], [29], [30], [31], [32], [33], [34], [35], [36], [37], [38], [39], [40], [41], [42], [43], [44], [45], [46], [47], [48], [49], [50], [51], [52], [53], [54]], followed by Mesoamerica (27%) [30,[55], [56], [57], [58], [59], [60], [61], [62], [63], [64], [65], [66], [67], [68], [69]], and only 1 in the Caribbean (2%) [70]; 18% of the studies used data collected across these subregions [[15], [16], [17],[71], [72], [73], [74], [75], [76], [77], [78]]. Of the eligible studies, 80% were conducted in upper-middle-income countries, and 72% collected data in both urban and rural settings. The studies had a median number of participants of 4379 (IQR, 2036–8170), with most of them including both sexes (92%), and covering a wide range of ages (73%), as many studies involved mother–child pairs.

**TABLE 1:** Characteristics of Eligible Studies Evaluating DBM in the LAC Region^1^

		All studies (*N* = 60)
Study details		
Publication year	2000–2007	8 (13.3)
	2008–2015	21 (35.0)
	2016–2023	31 (51.7)
Study design	Cross-sectional	57 (95.0)
	Cohort studies	3 (5.0)
Source	DHS only	15 (25.0)
	DHS + other	6 (10.0)
	National Nutritional Survey	12 (20.0)
	Other	27 (45.0)
National representativeness	Yes	41 (68.3)
	No	14 (23.3)
	Unspecified	5 (8.4)
Methodological quality	Low risk of bias	42 (70.0)
	Moderate risk of bias	18 (30.0)
	High risk of bias	0 (0.0)
Setting		
Subregion^2^	Mesoamerica	16 (26.7)
	South America	32 (53.3)
	Caribbean	1 (1.7)
	Mixed regions	11 (18.3)
Country income^3^	Low-middle-income	2 (3.3)
	Upper-middle-income	48 (80.0)
	High-income	1 (1.7)
	Mixed incomes	9 (15.0)
Type of area	Urban	7 (11.7)
	Rural	2 (3.3)
	Rural and urban	43 (71.7)
	Semirural/semiurban	2 (3.3)
	Unspecified	6 (10.0)
Population		
Total participants^4^	Number	4379 (2036–8170)
Gender	Male	0 (0.0)
	Female	5 (8.3)
	Both	55 (91.7)
Age group (y)	<5	7 (11.7)
	5–12	0 (0.0)
	13–18	3 (5.0)
	18–60	2 (3.3)
	>60	3 (5.0)
	Multiple ages	44 (73.3)
	Pregnant women	1 (1.7)

Abbreviation: DHS, Demographic and Health Survey.

1 Values are *n* (%) or median (IQR).

2 Countries belonging to each subregion were as follows: Mesoamerica—Belize, Costa Rica, Guatemala, Honduras, Mexico, Nicaragua, and El Salvador; South America—Argentina, Bolivia, Brazil, Chile, Colombia, Ecuador, Guyana, Peru, Paraguay, Suriname, and Uruguay; Caribbean—Barbados, Cuba, Domenica, Dominican Republic, Haiti, British Virgin Island, Jamaica, Saint Kitts and Nevis, and Saint Lucia.

3 The countries belonging to each country income category were as follows: Low-middle—Bolivia and Haiti; upper-middle—Argentina, Brazil, Colombia, Ecuador, Guatemala, Mexico, and Peru; high-income—Uruguay; mixed income—Argentina, Barbados, Belize, Bolivia, Brazil, British Virgin Islands, Chile, Colombia, Costa Rica, Cuba, Dominican Republic, Dominica, El Salvador, Guatemala, Guyana, Haiti, Honduras, Jamaica, Mexico, Nicaragua, Paraguay, Peru, Saint Kitts and Nevis, Saint Lucia, Suriname, and Uruguay.

4 Total participants are based on the number of 364 estimates.

From the 17 DBM typologies identified, descriptive information on 387 estimates could be extracted representing 27 different countries in LAC region (Table 2). The countries with the highest number of estimates were Peru (70 estimates), Brazil (46), and Mexico (44). Overall, the estimates used data collected between 1988 and 2017. From this, 41% of estimates used data collected between 2001 and 2011, 31% between 2012 and 2017, and 27% between 1988 and 2000. Among children, the most frequently evaluated type of undernutrition was stunting (70%), whereas the type of overnutrition was overweight (100%). For adults, the most frequent types of undernutrition were underweight and anemia (both 38%), and the most common type of overnutrition was overweight (98%) according to BMI (kg/m^2^). Regarding the levels and definitions of DBM, the majority of household DBM used the definition of adult with overnutrition + child with undernutrition and at the individual level, most were related to the definition of adult with overnutrition + undernutrition. Only 1% of the studies assessed DBM across the life course. Adults in most studies related to women; only 10 of 387 estimates referred to both sexes, men and women, and none to men alone.

**TABLE 2:** Specifications of DBM Estimates^1^

		LAC	Mesoamerica^2^	South America^2^	Caribbean^2^
		Estimates = 387	Estimates =113	Estimates = 225	Estimates = 49
Data collection details					
Year of data collection	1988–2000	104 (26.9)	29 (25.7)	57 (25.3)	18 (36.7)
	2001–2011	160 (41.3)	45 (39.8)	108 (48.0)	7 (14.3)
	2012–2017	119 (30.8)	39 (34.5)	56 (24.9)	24 (49.0)
	Unspecified	4 (1.0)	0 (0.0)	4 (1.8)	0 (0.0)
The contemporaneity of data collection	Yes	377 (97.4)	113 (100.0)	215 (95.6)	49 (100.0)
	No	2 (0.5)	0 (0.0)	2 (0.9)	0 (100.0)
	Unspecified	8 (2.1)	0 (0.0)	8 (3.5)	0 (100.0)
Types of malnutrition independently^3^					
Type of undernutrition in children (<18 y)	Stunting by HA	225 (70.3)	65 (73.9)	124 (66.0)	36 (81.8)
	Wasting by WA or WH	34 (10.6)	11 (12.5)	17 (9.0)	6 (13.6)
	Underweight by WA or BMI-for-age	6 (1.9)	1 (1.1)	5 (2.7)	0 (0.0)
	Anemia by laboratory	32 (10.0)	11 (12.5)	19 (10.1)	2 (4.6)
	Other micronutrient deficiencies^4^	6 (1.9)	0 (0.0)	6 (3.2)	0 (0.0)
	Mixed^6^	18 (5.3)	0 (0.0)	17 (9.0)	1 (0.0)
Type of overnutrition in children (<18 y)	Overweight by WH or BMI-for-age	139 (100.0)	44 (100.0)	76 (100.0)	19 (100.0)
Type of undernutrition in adults (>18 y)	Underweight by BMI	24 (37.5)	9 (36.0)	11 (31.4)	4 (100.0)
	Short stature by height cutoff	7 (10.9)	3 (12.0)	4 (11.4)	0 (0.0)
	Anemia	24 (37.5)	10 (40.0)	14 (40.0)	0 (0.0)
	Other micronutrient deficiencies^5^	9 (12.1)	3 (12.0)	6 (17.1)	0 (0.0)
Type of overnutrition in adults (>18 y)	Overweight by BMI	231 (98.0)	66 (95.6)	145 (98.6)	30 (100.0)
	Central obesity by waist-related indicators^7^	5 (2.0)	3 (4.4)	2 (1.4)	0 (0.0)
DBM details					
Level of assessment	Individual	155 (40.1)	51 (45.1)	89 (39.6)	15 (30.6)
	Household/pair	229 (59.2)	60 (53.1)	135 (60.0)	34 (69.4)
	Across the life course	3 (0.7)	2 (1.8)	1 (0.4)	0 (0.0)
Definition of DBM^8^	Child with overnutrition + undernutrition	115 (29.7)	34 (30.1)	66 (29.3)	15 (30.6)
	Adult with overnutrition + undernutrition	41 (10.6)	17 (15.0)	24 (10.7)	0 (0.0)
	Adult with overnutrition + child with undernutrition	205 (53.0)	52 (46.0)	123 (54.7)	30 (61.2)
	Child with overnutrition + adult with undernutrition	25 (6.5)	10 (8.9)	11 (4.9)	4 (8.2)

Abbreviations: HA, height-for-age; WA, weight-for-age; WH, weight-for-height.

1 Values are *n* (%).

2 The regions included the following countries and number of estimates: Mesoamerica—Belize (3), Costa Rica (1), Guatemala (30), Honduras (17), Mexico (44), Nicaragua (16), and El Salvador (2); South America—Argentina (4), Bolivia (28), Brazil (46), Chile (1), Colombia (39), Ecuador (18), Guyana (10), Peru (70), Paraguay (2), Suriname (3), and Uruguay (4); Caribbean—Barbados (1), Cuba (1), Domenica (1), Dominican Republic (19), Haiti (23), British Virgin Island (1), Jamaica (1), Saint Kitts and Nevis (1), and Saint Lucia (1).

3 Types of malnutrition independently do not add to the total estimates per column, as the total estimates per column are calculated based on DBM estimates.

4 Other micronutrient deficiencies in children included the following: zinc deficiency (3), selenium deficiency (1), copper deficiency (1), and vitamin A deficiency (1).

5 Other micronutrient deficiencies in adults included the following: zinc deficiency (3), vitamin A deficiency (2), vitamin B-12 deficiency (2), and folate deficiency (2).

6 Mixed indicates the following combination of types of undernutrition: stunting + underweight (5), stunting + anemia (2), wasting + underweight (5), wasting + stunting + underweight (6).

7 Waist-related indicators included waist circumference (3), waist-to-hip ratio (1), and waist-to-height ratio (1).

8 Overnutrition includes overweight, obesity, and central obesity, undernutrition includes stunting, wasting, underweight, short stature, anemia, and other micronutrient deficiency.

From the 387 DBM typology estimates, 360 estimates had the required information to be included in meta-analyses. Of these, the 3 most common typologies were adult with overweight + child with stunting (37%), child with overweight + stunting (24%), and adult with overweight + child with wasting (9%). The frequency of the DBM typologies estimates by the LAC country is presented in Supplemental Figure 1.

**FIGURE 3:**
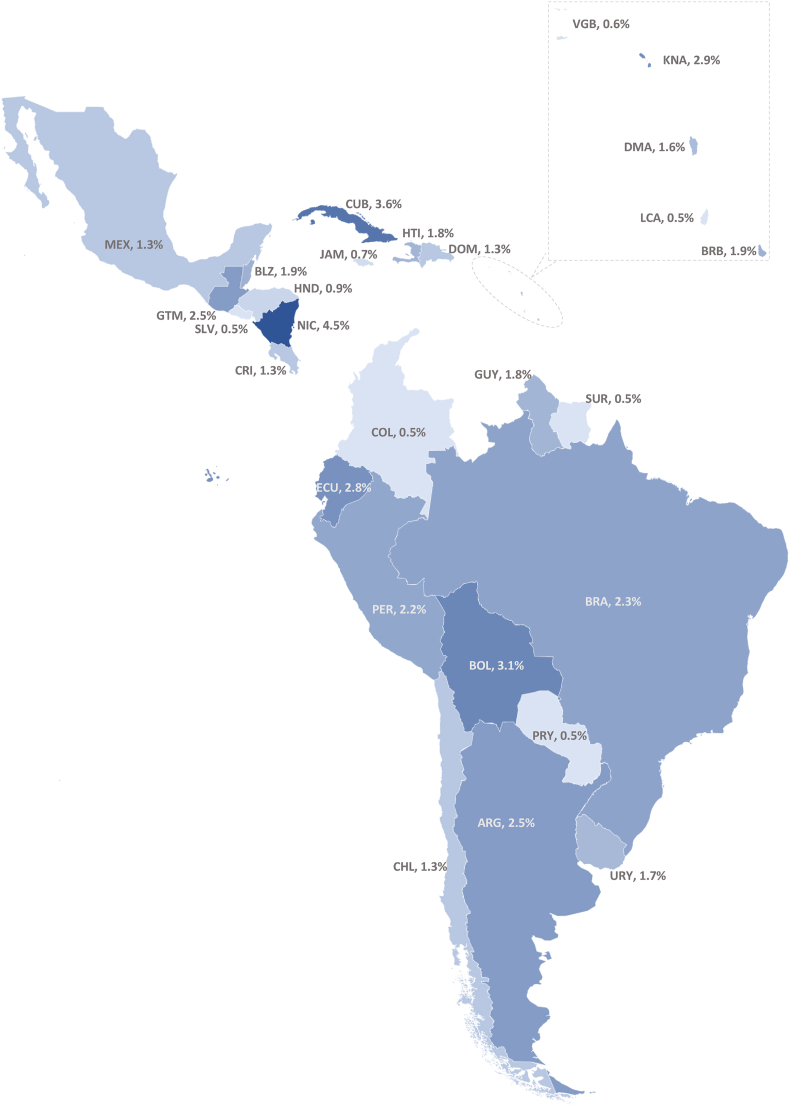
Pooled period prevalence of DBM typology child (younger than 18 years) with overweight + stunting by country covering 1988–2017. For each country, the pooled period prevalence with the corresponding 95% CI was obtained using a random-effect meta-analysis of proportions (command *metaprop*). Freeman–Tukey double arcsine transformation was used to stabilize variances from extreme proportional estimates. Each country had a different number of DBM estimates: Argentina (4), Barbados (1), Belize (3), Bolivia (28), Brazil (43), British Virgin Islands (1), Chile (1), Colombia (36), Costa Rica (1), Cuba (1), Dominican Republic (18), Dominica (1), Ecuador (12), El Salvador (2), Guatemala (30), Guyana (10), Haiti (23), Honduras (17), Jamaica (1), Mexico (33), Nicaragua (15), Paraguay (2), Peru (68), Saint Kitts and Nevis (1), Saint Lucia (1), Suriname (3), and Uruguay (4).

**FIGURE 4:**
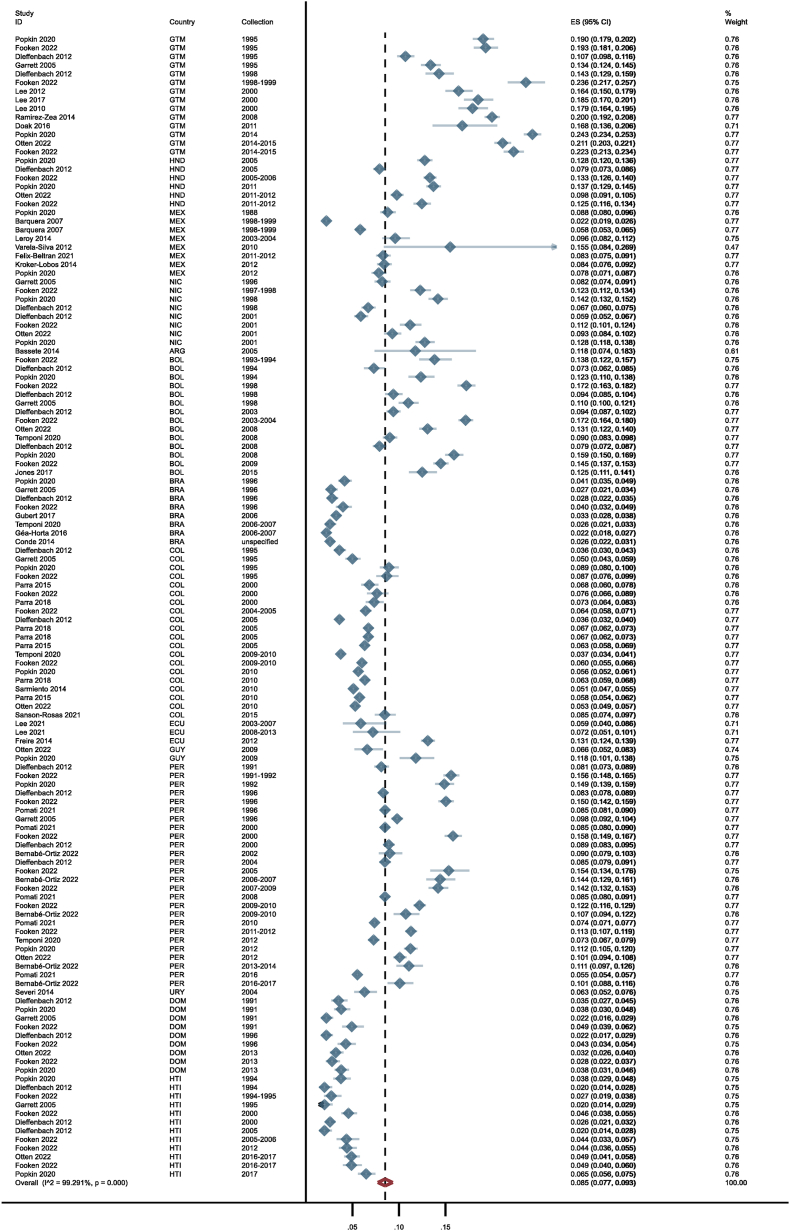
Forest plot: pooled period prevalence for DBM typology adult (older than 18 years) with overweight + child (younger than 18 years) with stunting.

Table 3 presents the prevalence of the identified DBM typologies between 1988 and 2017. At the individual level for children, estimated prevalences included 1.6% (0.016; 95% CI: 0.016 0.017) for overweight + stunting after pooling 88 estimates (Figure 3 illustrates the prevalence for this typology by country), 3.6% (0.036; 0.021, 0.054) for overweight + anemia using 20 estimates, and 11.5% (0.115; 0.073, 0.165) for overweight + other micronutrient deficiencies, after pooling 5 estimates. Likewise, at the individual level for adults, prevalences of 21.6% (0.216; 0.140, 0.304) were observed for overweight + short stature based on 5 estimates and 6.9% (0.069; 0.042, 0.103) for overweight + anemia after pooling 12 estimates. At the household level, we found the following prevalence figures: 8.5% (0.085; 0.077, 0.093) for adult with overweight + child with stunting based on 132 estimates (Figure 4), 0.8% (0.008; 0.006, 0.011) for adult with overweight + child with wasting (34 estimates), 0.0% (0.000; 0.000, 0.001) for adult with overweight and child with underweight (5 estimates), 24.3% (0.243; 0.188, 0.302) for adult with overweight + child with anemia (10 estimates), 0.7% (0.0007; 0.001, 0.019) for adult with overweight + child with mixed undernutrition (17 estimates), and 0.2% (0.002; 0.001, 0.003) for child with overweight + adult with underweight (23 estimates). Forest plots for all outcomes are illustrated in Supplemental Figures 2–11. For the outcomes of child with overweight + mixed undernutrition, adult with central obesity + short stature, adult with overweight + other micronutrient deficiencies, adult with central obesity + underweight, adult with overweight + child with other micronutrient deficiencies, and adult with central obesity + child with stunting, no sufficient data could be retrieved to conduct meta-analyses.

**TABLE 3:** Pooled Period Prevalence for All DBM Typologies in the LAC Region Covering 1988–2017

	No. of estimates	No. of participants, median (IQR)	Pooled period prevalence (95% CI)^1^	Prevalence (%)	*I*^2^ (%)	Heterogeneity *P*
DBM typologies at the individual level						
Children 0–18 y with overnutrition + undernutrition						
Overweight + stunting	88	4300 (2171–9664)	0.016 (0.016, 0.017)	1.6 (1.6–1.7)	99.6	<0.001^2^
Overweight + anemia	20	3723 (1031–7918)	0.036 (0.021, 0.054)	3.6 (2.1–5.4)	99.5	<0.001^2^
Overweight + other micronutrient deficiencies^3^	5	148 (148–1524)	0.115 (0.073, 0.165)	11.5 (7.3–16.5)	94.5	<0.001^2^
Overweight + mixed undernutrition^4^	1	NA	NA	NA	NA	NA
Any DBM at this level^5^	114	3787 (1896–8573)	0.019 (0.018, 0.020)	1.9 (1.8–2.0)	99.6	<0.001^2^
Adults >18 y with overnutrition + undernutrition						
Overweight + short stature	5	532 (446–1308)	0.216 (0.140, 0.304)	21.6 (14.0–30.4)	98.1	<0.001^2^
Central obesity + short stature	2	NA	NA	NA	NA	NA
Overweight + anemia	12	4526 (1102–9385)	0.069 (0.042, 0.103)	6.9 (4.2–10.3)	99.6	<0.001^2^
Overweight + other micronutrient deficiencies^6^	2	NA	NA	NA	NA	NA
Central obesity + underweight	1	NA	NA	NA	NA	NA
Any DBM at this level^5^	22	1699 (761–7205)	0.112 (0.077, 0.152)	11.2 (7.7–15.2)	99.7	<0.001^2^
DBM typologies at the household level						
Adult with overnutrition + child with undernutrition						
Overweight + stunting	132	3967 (2035–7123)	0.085 (0.077, 0.093)	8.5 (7.7–9.3)	99.3	<0.001^2^
Overweight + wasting	34	4701 (2688–7970)	0.008 (0.006, 0.011)	0.8 (0.6–1.0)	97.7	<0.001^2^
Overweight + underweight	5	14,812 (11,565–26,805)	0.000 (0.000, 0.001)	0.0 (0.0–0.1)	95.0	<0.001^2^
Overweight + anemia	10	2219 (1042–7025)	0.243 (0.188, 0.302)	24.3 (18.8–30.2)	99.3	<0.001^2^
Overweight + other micronutrient deficiencies^7^	1	NA	NA	NA	NA	NA
Central obesity + stunting	2	NA	NA	NA	NA	NA
Overweight + mixed undernutrition^8^	17	14,812 (11,565–26,805)	0.007 (0.001, 0.019)	0.7 (0.1–1.9)	99.9	<0.001^2^
Any DBM at this level^5^	201	4530 (2225–8189)	0.060 (0.051, 0.070)	6.0 (5.1–7.0)	99.9	<0.001^2^
Child with overnutrition + adult with undernutrition						
Overweight + underweight	23	4486 (2688–7242)	0.002 (0.001, 0.003)	0.2 (0.1–0.3)	87.7	<0.001^2^

1 Pooled period prevalence with corresponding 95% CI were obtained using a random-effect meta-analysis of proportions (command *metaprop*). Freeman–Tukey double arcsine transformation was used to stabilize variances from extreme proportional estimates.

2 Results indicate statistical significance for heterogeneity based on an α of 0.05.

3 Other micronutrient deficiencies in children included the following: zinc deficiency (2), selenium deficiency (1), copper deficiency (1), and vitamin A deficiency (1).

4 Mixed undernutrition indicates: anemia + stunting (1).

5 Any DBM refers to the pooling of all typologies within the specific level (individual/household) and population (children 0–18, adults >18, and adult–child pair). For the typologies related to child with overnutrition + adult with undernutrition, any DBM could not be estimated because only 1 typology (child with overweight + adult with underweight) was available.

6 Other micronutrient deficiencies in adults included zinc deficiency (1) and vitamin A deficiency (1).

7 Other micronutrient deficiencies at the household level indicate zinc deficiency (1).

8 Mixed undernutrition indicates the following combination of types of undernutrition: stunting + underweight (5), stunting + anemia (1), wasting + underweight (5), and wasting + stunting + underweight (6).

The prevalence for all DBM typologies showed substantial levels of heterogeneity (*I*^2^ > 75%). The meta-regressions used to explore potential sources of heterogeneity for DBM typologies are shown in Supplemental Tables 4–6. For the outcome of adult with overweight + child with stunting, subregions and countries were identified as potential heterogeneity sources. Considering only the estimates collected in the Caribbean resulted in a modest decrease in heterogeneity (stratified prevalence: 3.5%; *I*^2^: 88.6%, vs nonstratified prevalence: 8.5%; *I*^2^: 99.3%). When stratifying by country, notable reductions were seen particularly for Brazil (3.0%; *I*^2^: 78.5%) and the Dominican Republic (3.3%; *I*^2^: 79.0%) compared with the nonstratified prevalence for this outcome (8.5%; *I*^2^: 99.3%). Although other sources of heterogeneity were identified in the DBM typologies adult with overweight + anemia and child with overweight + stunting, the level of heterogeneity slightly decreased but remained high in the stratified meta-analyses (Supplemental Table 7).

The stratified meta-analyses based on categories of year of data collection for 2 outcomes are presented in Supplemental Table 8. These analyses provide an insight into the evolution of the prevalences suggesting that although the typology child with overweight + stunting seems to be decreasing, the typology adult with overweight + child with stunting is increasing over time.

The sensitivity analyses based on nationally representative estimates (Supplemental Table 9) showed a decrease in the prevalence estimates, varying between 0.1% and 0.9%, for most DBM typologies. Notable changes were found for the typologies of an adult with overweight + anemia, with an increase of 0.9% in the prevalence, and for adult with overweight + child with anemia, with a prevalence decrease of 4.2%. Heterogeneity levels remained high (*I*^2^ > 85%) regardless of the use of nationally representative data only.

We identified only 2 prospective studies that evaluated DBM across the life course. Jones-Smith et al. [60] observed that Mexican infants with a small size at birth and who had accelerated growth in the first year had 3.58 higher odds of obesity at the age of 4–6 years than those who did not have accelerated growth independently of the size at birth [60]. In Brazil, Lourenço et al. [29] evaluated the influence of sociodemographic and maternal and child early determinants on the growth trajectories of BMI-for-age Z scores using longitudinal data from 255 children and observed that infants who had a low birth weight (<2500 g) or who were stunted at birth had a normal BMI-for age *z*-score at the age of 10, indicating no overweight or obesity [29]. No DBM prevalence across the life course was available from these 2 studies.

## Discussion

This systematic review synthesized information from 60 studies that estimated DBM at the household, individual, or across the life course levels in the LAC region and were published over the last 2 decades. Using information from 360 estimates of the 17 DBM typologies identified in the literature, we found that the prevalence of DBM ranges between 0% and 24% in the LAC region.

From the evidence retrieved, it was observed that although a large number of studies have been conducted to evaluate DBM in countries such as Peru, Brazil, and Mexico, evidence is not yet available for other countries such as Panama or Venezuela. Moreover, there is a data gap between 2018 and 2023 because the data derived from the eligible evidence only covers the period from 1988 to 2017. Similarly, very few studies included men or older adults (>65 years). Furthermore, most estimates were derived from household-level studies and only a few from studies at the individual level, implying that specific vulnerable groups of individuals (e.g., prisoners and migrants) are not included in the evidence. Woman with overweight + child with stunting was commonly reported in households, with a worrying 8.5% of households across LAC region being affected by this typology. Individually, child with excess weight and stunting, and woman with overweight and anemia were also frequently reported with a prevalence of 2% and 7%, respectively. Importantly, the typologies with the highest prevalence of DBM were those based on a small number of estimates drawn from a few studies. These typologies included adult with overweight + child with anemia, with a prevalence of 24%; child with overweight + other micronutrient deficiencies, 12%; and adult with overweight + short stature, 22%. Insufficient evidence was available to evaluate DBM across the life course level, likely due to the need for longitudinal data and extended follow-up time. Common components observed across DBM typologies included a high prevalence of overweight in adults, as well as anemia and other micronutrient deficiencies in both children and adults, hence suggesting that these components contribute substantially to the occurrence of DBM in this region. Nevertheless, we must also remain critical of these findings as to whether the observed DBM prevalences are a result of available evidence or whether they accurately represent the true state of DBM. In particular, the fact that the typologies with the highest prevalence often have fewer estimates complicates the interpretation of these findings. Furthermore, our study has highlighted the need for further data on potential micronutrient deficiencies as a component of DBM and for more information on understudied populations such as men and particularly older people, among which malnutrition is highly prevalent [79], and the consequences of DBM more significant. Information on the DBM at the life course level needs also to be explored because it will help in understanding the epidemiology of a prospective accumulation of malnutrition types throughout the lifespan.

Studies estimating the prevalence of specific typologies of DBM in the LAC region are available. For children younger than 5 years, a prevalence of 0.5%–0.6% for overweight + stunting was reported [16,76], rising to 1.9% for those between 12 and 15 years [72], whereas we found an estimated prevalence of 1.6% for those younger than 18 years. Similarly, a study using DHS data estimated a prevalence of 10.4% for adult with overweight + child with stunting, 0.5% for adult with overweight + child with wasting, and 19.4% for adult with overweight + child with anemia [15], showing slight variations with our estimates, 8.5%, 0.8%, and 24.3%, respectively. These differences are potentially explained by the variations in age cutoffs and the number of estimates used coming from several data sources.

Comparing our findings with other regions highly affected by DBM, the Middle East and North African region seem to have a modestly higher prevalence for child with overweight + stunting, ranging between 2.4% and 3.4% among infants of 5 years and adolescents [16,72,76] than the one estimated for the LAC region (1.6%). Moreover, when compared with a prevalence of 7% for adult (mainly women) with overweight + anemia observed for the LAC region, the African and Eastern Mediterranean regions seem to have remarkably higher prevalence figures, 11% and 14%, respectively [80]. For the typology adult with overweight + child with stunting, the South and South East Asia region (10%) [81], seems to have a slightly higher prevalence than the LAC region (8.5%).

It is well-known that DBM is partly interlinked with progressing stages of the economic, demographic, and epidemiological transition. More importantly, it is a hallmark of the nutrition transition, away from famine patterns, toward rapid access to and adoption of suboptimal diets and lifestyles, leading to changes in nutritional status [12]. In addition to these transitions, the LAC region is widely known for having a high proportion of health inequalities, particularly social and economic inequalities [82]. These disparities are interlinked with disrupting food systems, particularly challenging food security [83]. In this line, evidence has highlighted the relation between the current food systems, particularly the ubiquitous presence of ultraprocessed foods, and DBM [84]. A recent study in Mexico evaluated the association between ultraprocessed foods and DBM, defined as the coexistence of overweight and anemia in children and adolescents, and observed that these foods increased the odds of DBM by 30% in children from a lower socioeconomic status and by 26% in adolescents from a higher socioeconomic status [65]. Of concern, ultraprocessed foods seem to play a role not only in the development of overweight and lifestyle-related NCD but also in stunting, which is a result of providing nutrient-depleted calories during the first 1000 d of life [77]. In this line, a meta-analysis showed that the consumption of these foods correlates with diets of low nutritional quality such as low in fiber and essential vitamins and minerals [85]. These results might explain the high prevalence of DBM related to overweight, anemia, and other micronutrient deficiencies observed in our findings. We encourage future research to understand and enhance strategies that address overweight, including the promotion of high-nutritional-quality diets among the general population taking into account context-based inequalities. This will enable substantial progress to be made in curbing the burden of DBM in the LAC region.

DBM comes along with a sizeable economic burden in a country. In Ecuador, the economic impact of DBM equals 4.3% of the gross domestic product corresponding to United States $4.3 billion per year, whereas in Mexico, it reaches 2.3% (United States $ 28.8 billion per year) [86]. In response to the complexity of DBM, the WHO has suggested double-duty actions, which are defined as interventions that have the potential to simultaneously reduce the burden of undernutrition, obesity, or diet-related NCDs based on the principle of tackling shared drivers [87]. The Lancet Commission also proposed holistic actions to be implemented in health services, educational settings, agriculture, and food systems and environments, among others, such as scale-up programs for breastfeeding, food subsidies and vouchers, school feeding programs, and agricultural, and food system policies for healthy diets [88]. However, the impact of suggested actions appears to be limited [89,90]. Our findings add to the body of evidence [[91], [92], [93]] calling for action from policymakers to invest in the development and implementation of contextually appropriate public health strategies to address DBM considering multidisciplinary approaches (e.g., socioecological models or frameworks) [94,95], which if not urgently addressed will continue to pose harm to populations and countries development.

### Strengths and limitations

To our knowledge, this is the first study to provide a comprehensive overview of the evidence evaluating DBM, by compiling information from 60 studies, at household, individual, and across life course levels in the LAC region. Using innovative approaches and definitions and several data sources, this study summarizes >300 estimates of DBM and provides important insights into the prevalence of DBM and its respective typologies at different levels. Moreover, the majority of the evidence included used nationally representative data and was graded as having a low risk of bias. However, our study is not free of limitations. As a common disadvantage of meta-analyses of proportions, our prevalence estimates show large levels of heterogeneity regardless of heterogeneity exploration using meta-regressions and stratified and sensitivity analyses. Moreover, the method used to collect and diagnose malnutrition outcomes was not considered because this was out of the scope of this study. Nevertheless, this could have influenced the comparability of outcomes. Our findings, including DBM prevalence estimates, may not be generalizable to every setting of LAC region because bias could have been introduced owing to limited or unavailable data for specific settings and/or typologies.

### Conclusion

This systematic review and meta-analysis show that, based on the available data, the prevalence of DBM ranges between 0% and 24% in the LAC region, the magnitude of DBM strictly depends on the DBM typology. Our findings do not imply that the remaining population is absent of malnutrition outcomes because they may present them independently (e.g., only obesity) or even as a triple burden of malnutrition. Adults, particularly women owing to data availability, with overweight appear to be an important contributor to DBM; hence, strategies aimed addressing overweight in this group could result in substantial improvements in DBM. We encourage further research on DBM to prioritize understudied populations, assess DBM across the life course, and incorporate micronutrient deficiency as a component of DBM. This information is key to producing robust estimates of DBM so that public health decision-makers can take immediate action to combat DBM.

## Author contributions

The authors’ responsibilities were as follows – DS, JLP: developed the research protocol and methodology; DS, AB-P: developed the data extraction tools; DS, AB-P, AR-A, JLP: reviewed literature, selected eligible studies, and performed data extractions; DS: conducted the statistical analyses under the supervision of JLP; DS, JLP: interpreted results and drafted the manuscript; DS, AB-P, AR-A, KP, MR-Z, LB, JLP: reviewed and edited the manuscript. DS, AB-P, JLP: accessed and verified the data; DS, JLP are responsible for the decision to submit the manuscript for publication; and all authors: reviewed and approved the final manuscript.

## Conflict of interest

The authors report no conflicts of interest.

## Funding

This study was funded by internal funds of the Department of Public Health, Institute of Tropical Medicine, Antwerp, Belgium.

## Data availability

The data generated in this study may be accessible under request by contacting the corresponding author: DS (dsagastume@itg.be).

## Appendix A Supplementary data

Multimedia component1 Multimedia component1
