# Supplementary material for: Quantifying Overlapping Forms of Malnutrition Across Latin America: A Systematic Literature Review and Meta-Analysis of Prevalence Estimates
Source: Adv Nutr. 2024 Mar 15;15(5):100212. doi: 10.1016/j.advnut.2024.100212 (PMC11015105; doi:10.1016/j.advnut.2024.100212)
Supplement: Multimedia component1 [file mmc1.pdf]

Supplementary material

Quantifying overlapping forms of malnutrition across Latin America: A Systematic Literature Review and Meta-Analysis of Prevalence Estimates

Diana Sagastume, MSc[1,2] *, Antonio Barrenechea-Pulache, MD[3], Andrea Ruiz-Alejos, MD[2], Prof Katja Polman, PhD[1,4], Prof Lenka Beňová, PhD[1] , Manuel Ramírez-Zea, PhD[5], Prof José L Peñalvo, PhD[2,6]

Affiliations

[1] Department of Public Health, Institute of Tropical Medicine, Antwerp, Belgium

[2] Global Health Institute, University of Antwerp, Wilrijk, Belgium

[3] Universidad Científica del Sur, Lima, Perú

[4] Department of Health Sciences, Vrije Universiteit (VU) Amsterdam, The Netherlands

[5] INCAP Research Center for the Prevention of Chronic Diseases (CIIPEC), Institute of Nutrition of Central America and Panama (INCAP), Guatemala City, Guatemala

[6] National Center for Epidemiology, Carlos III Institute of Health (ISCIII), Madrid, Spain

***Correspondence:**

Diana Sagastume, Institute of Tropical Medicine, Nationalestraat 155, 2000 Antwerp, Belgium. Email: dsagastume@itg.be

Content

Section 1: Methodology 3

1.1 Deviations from the original protocol 3

1.2 Systematic search 3

1.3 Exclusion criteria 5

1.4 Extracted data 5

Table S1. List of assumptions of eligible studies 5

Table S2. DBM typologies identified and included 7

Section 2: Results 8

Table S3. Evidence table of eligible studies 8

Figure S1. Heat map of the frequency of DBM typologies by country 11

Figure S2. Forest plot – Individual level in children 0-18 years, typology overweight + stunting 12

Figure S3. Forest plot – Individual level in children 0-18 years, typology overweight + anemia 12

Figure S4. Forest plot – Individual level in children 0-18 years, typology overweight + other micronutrient deficiencies 13

Figure S5. Forest plot – Individual level in adults >18 years, typology overweight + short stature 13

Figure S6. Forest plot – Individual level in adults >18 years, typology overweight + anemia 14

Figure S7. Forest plot – Household level, adult with overweight + child with wasting 14

Figure S8. Forest plot – Household level, adult with overweight + child with underweight 15

Figure S9. Forest plot – Household level, adult with overweight + child with anemia 15

Figure S10. Forest plot – Household level, adult with overweight + child with mixed undernutrition 16

Figure S11. Forest plot – Household level, child with overweight + adult with underweight 16

Table S4. Univariate meta-regressions: Heterogeneity exploration for the outcomes related to DBM typologies at the individual level ... 17

Table S5. Univariate meta-regressions: Heterogeneity exploration for the outcomes related to DBM typologies at the household/pair level 19

Table S6. Multivariate meta-regressions: Heterogeneity exploration for the outcomes related to DBM typologies at the individual and household/pair level 21

Table S7. The pooled period prevalence of DBM typologies stratified by the encountered source of heterogeneity covering 1998-2017 . 22

Table S8. Stratified analysis – Pooled period prevalence of DBM typologies stratified by year of data collection 23

Table S9. Sensitivity analysis – Pooled period prevalences of DBM typologies limited to nationally representative data covering 1998-2017 24

References 25

Section 1: Methodology

1.1 Deviations from the original protocol

The protocol of this study was registered in the PROSPERO database (CRD42023406755). Deviations from the initial protocol include the following:

- Due to the large amount of evidence found to answer research question 1 ‘What is the prevalence of DBM, in its multiple combinations, at the household, individual, and across the life course levels in Latin America?’ the investigators considered to limit this manuscript to only answer this research question. We expect to publish another manuscript answering research question #2 ‘To what extent modifiable drivers of DBM have been investigated and are there strategies/interventions for DBM in place?’ as this information has been extracted already.
- Due to the large amount of scientific evidence and extracted estimates derived from the literature databases only, grey literature including international agencies' technical reports/repositories of international health agencies, for instance, the Pan American Health Organization were only used for discussion purposes.
- We decided to not define primary or secondary outcomes anymore and considered all the DBM typologies identified in the literature as outcomes. This decision was made after considering all available evidence and to provide the same relevance and importance to all DBM typologies, independently of the DBM level.
- Sensitivity analyses of pooled prevalences using only nationally representative data were not considered in the initial protocol.

1.2 Systematic search

<u>PubMed (289 hits):</u>

Specifications:

- 2000-2023

Search strategy

- ((((("Overweight"[MAJR] AND "epidemiology" [Subheading]) AND ("Micronutrient*"[MAJR] AND "deficiency" [Subheading]))) OR (("double burden" OR "dual burden" OR "triple burden" OR "coexistence" OR "co-existence") AND nutrition*)) OR ((("double burden"[ti] OR "dual burden"[ti] OR "triple burden"[ti] OR "coexistence"[ti] OR "co-existence"[ti] AND (malnutrition or undernutrition))) OR ((((((("Overweight"[Majr] AND "epidemiology" [Subheading]) AND ("Micronutrients"[MAJR] AND "deficiency" [Subheading]))) OR (("double burden" OR "dual burden" OR "triple burden") AND nutrition*)) OR ((overweight OR obesity OR obese OR overnutrition) AND (micronutrient* OR anemia* OR stunt* OR underweight OR "short stature" OR "short height" OR "undernutrition" OR "undernourished")))) AND ((("obesity/prevention and control"[MAJR] AND "humans"[Mesh])) OR ((Normal Body Weight[tw] OR "body mass index"[MeSH Major Topic]) AND ("diet"[Mesh] OR "exercise"[Mesh]) NOT "weight gain/drug effects"[Mesh])))) AND ("Latin America"[Mesh] OR "Latin America*" OR "Hispanic or Latino"[Mesh] OR latin* OR "Antigua and Barbuda"[Mesh] OR "Antigua and Barbuda*" OR "Argentina"[Mesh] OR "Argentin*" OR "Aruba"[Mesh] OR "Aruba*" OR "Bahamas"[Mesh] OR "Baham*" OR "Barbados"[Mesh] OR "Barbad*" OR "Belize"[Mesh] OR "Belize*" OR "Bolivia"[Mesh] OR "Bolivia*" OR "Brazil"[Mesh] OR "Brazil*" OR "British Virgin Islands"[Mesh] OR "British Virgin Island*" OR "West Indies"[Mesh] OR "West Indies" OR "Cayman Island*" OR "Chile"[Mesh] OR "Chile*" OR "Colombia"[Mesh] OR "Colombia*" OR "Costa Rica"[Mesh] OR "Costa Rica*" OR "Cuba"[Mesh] OR "Cuba*" OR "Curacao"[Mesh] OR "Curacao" OR "Curaçao" OR "Dominica"[Mesh] OR "Dominica*" OR "Dominican Republic"[Mesh] OR "Dominican Republic" OR "Dominican*" OR "Ecuador"[Mesh] OR "Ecuador*" OR "El Salvador"[Mesh] OR "salvadoran*" OR "Grenada"[Mesh] OR "Grenad*" OR "Guatemala"[Mesh] OR "Guatemala*" OR "Guyana"[Mesh] OR "Guyan*" OR "Haiti"[Mesh] OR "Haiti*" OR "Honduras"[Mesh] OR "Hondura*" OR "Jamaica"[Mesh] OR "Jamaica*"[Mesh] OR "Mexico"[Mesh] OR "Mexic*" OR "Nicaragua"[Mesh] OR "Nicaragua*" OR "Panama"[Mesh] OR "Panama*" OR "Paraguay"[Mesh] OR "Paraguay*" OR "Peru"[Mesh] OR "Peru*" OR "Puerto Rico"[Mesh] OR "Puerto Rica*" OR "Sint Maarten"[Mesh] OR "Sint Maarten" OR "St. Maarten" OR "Saint Martin" OR "St. Martin" OR "Saint Kitts and Nevis"[Mesh] OR "Saint Kitts and Nevis" OR "St. Kitts and Nevis" OR "Saint Lucia"[Mesh] OR "Saint Lucia" OR "St. Lucia" OR "Saint Vincent and the Grenadines"[Mesh] OR "Saint Vincent and the Grenadines" OR "St. Vincent and the Grenadines" OR" Suriname"[Mesh] OR "Suriname*" OR "Trinidad and Tobago"[Mesh] OR "Trinidad and Tobago" OR "Turks and Caicos Islands" OR "Uruguay"[Mesh] OR "Uruguay*" OR "Venezuela"[Mesh] OR "Venezuela*" OR "United States Virgin Islands"[Mesh] OR "United States Virgin Island*"))

<u>Web of Science (Hits 271)</u>

Specifications:

- Title search
- 2000/01/01 -2023/02/01

Search strategy:

- ((((("Overweight" AND "epidemiology") AND ("Micronutrient*" AND "deficiency"))) OR (("double burden" OR "dual burden" OR "triple burden" OR "coexistence" OR "co-existence") AND nutrition*)) OR ((("double burden" OR "dual burden" OR "triple burden" OR "coexistence" OR "co-existence" AND (malnutrition or undernutrition))) OR ((((((("Overweight" AND

"epidemiology") AND ("Micronutrients" AND "deficiency"))) OR (("double burden" OR "dual burden" OR "triple burden") AND nutrition*)) OR ((Overweight OR obesity OR obese OR overnutrition) AND (micronutrient* OR anemia* OR stunt* OR underweight OR "short stature" OR "short height" OR "undernutrition" OR "undernourished")))) AND ((("obesity/prevention and control" AND "humans")) OR ((Normal Body Weight[tw] OR "body mass index") AND ("diet" OR "exercise") NOT "weight gain/drug effects")))) AND ("Latin America" OR "Latin America*" OR "Hispanic or Latino" OR latin* OR "Antigua and Barbuda" OR "Antigua and Barbuda*" OR "Argentina" OR "Argentin*" OR "Aruba" OR "Aruba*" OR "Bahamas" OR "Baham*" OR "Barbados" OR "Barbad*" OR "Belize" OR "Belize*" OR "Bolivia" OR "Bolivia*" OR "Brazil" OR "Brazil*" OR "British Virgin Islands" OR "British Virgin Island*" OR "West Indies" OR "West Indies" OR "Cayman Island*" OR "Chile" OR "Chile*" OR "Colombia" OR "Colombia*" OR "Costa Rica" OR "Costa Rica*" OR "Cuba" OR "Cuba*" OR "Curacao" OR "Curacao" OR "Curaçao" OR "Dominica" OR "Dominica*" OR "Dominican Republic" OR "Dominican Republic" OR "Dominican*" OR "Ecuador" OR "Ecuador*" OR "El Salvador" OR "salvadoran*" OR "Grenada" OR "Grenad*" OR "Guatemala" OR "Guatemala*" OR "Guyana" OR "Guyan*" OR "Haiti" OR "Haiti*" OR "Honduras" OR "Hondura*" OR "Jamaica" OR "Jamaica*" OR "Mexico" OR "Mexic*" OR "Nicaragua" OR "Nicaragua*" OR "Panama" OR "Panama*" OR "Paraguay" OR "Paraguay*" OR "Peru" OR "Peru*" OR "Puerto Rico" OR "Puerto Rica*" OR "Sint Maarten" OR "Sint Maarten" OR "St. Maarten" OR "Saint Martin" OR "St. Martin" OR "Saint Kitts and Nevis" OR "Saint Kitts and Nevis" OR "St. Kitts and Nevis" OR "Saint Lucia" OR "Saint Lucia" OR "St. Lucia" OR "Saint Vincent and the Grenadines" OR "Saint Vincent and the Grenadines" OR "St. Vincent and the Grenadines" OR" Suriname" OR "Suriname*" OR "Trinidad and Tobago" OR "Trinidad and Tobago" OR "Turks and Caicos Islands" OR "Uruguay" OR "Uruguay*" OR "Venezuela" OR "Venezuela*" OR "United States Virgin Islands" OR "United States Virgin Island*"))

<u>Scopus (133 hits)</u>

Specifications:

- 2000 – 2023
- Articles and reviews

Search strategy:

- TITLE-ABS-KEY ((((("Overweight" AND "epidemiology") AND ("Micronutrient*" AND "deficiency"))) OR (("double burden" OR "dual burden" OR "triple burden" OR "coexistence" OR "co-existence") AND nutrition*)) OR ((("double burden" OR "dual burden" OR "triple burden" AND (malnutrition OR undernutrition))) OR ((((((("Overweight" AND "epidemiology") AND ("Micronutrients" AND "deficiency"))) OR (("double burden" OR "dual burden" OR "triple burden") AND nutrition*)) OR ((overweight OR obesity OR obese OR overnutrition) AND (micronutrient* OR anemia* OR stunt* OR underweight OR "short stature" OR "short height" OR undernutrition OR undernourished)))) AND ((("obesity prevention and control" AND "humans")) OR ((normal AND body AND weight OR "body mass index") AND ("diet" OR "exercise") not "weight gain drug effects")))) AND ("Latin America*" OR "Hispanic or Latino" OR latin* OR "Antigua and Barbuda*" OR "Argentin*" OR "Aruba*" OR "Baham*" OR "Barbad*" OR "Belize*" OR "Bolivia*" OR "Brazil*" OR "British Virgin Island*" OR "West Indies" OR "Cayman Island*" OR "Chile*" OR "Colombia*" OR "Costa Rica*" OR "Cuba*" OR "Curacao" OR "Curaçao" OR "Dominica*" OR "Dominican Republic" OR "Ecuador*" OR "El Salvador" OR "salvadoran*" OR "Grenad*" OR "Guatemala*" OR "Guyan*" OR "Haiti*" OR "Hondura*" OR "Jamaica*" OR "Mexic*" OR "Nicaragua*" OR "Panama*" OR "Paraguay*" OR "Peru*" OR "Puerto Rico" OR "Puerto Rica*" OR "Sint Maarten" OR "St. Maarten" OR "Saint Martin" OR "St. Martin" OR "Saint Kitts and Nevis" OR "St. Kitts and Nevis" OR "Saint Lucia" OR "St. Lucia" OR "Saint Vincent and the Grenadines" OR "St. Vincent and the Grenadines" OR "Suriname*" OR "Trinidad and Tobago" OR "Turks and Caicos Islands" OR "Uruguay*" OR "Venezuela*" OR "United States Virgin Island*")) AND (LIMIT-TO (EXACTKEYWORD , "Obesity") OR LIMIT-TO (EXACTKEYWORD , "Body Mass") OR LIMIT-TO (EXACTKEYWORD , "Body Mass Index") OR LIMIT-TO (EXACTKEYWORD , "Overweight")) AND (LIMIT-TO (PUBYEAR , 2023) OR LIMIT-TO (PUBYEAR , 2022) OR LIMIT-TO (PUBYEAR , 2021) OR LIMIT-TO (PUBYEAR , 2020) OR LIMIT-TO (PUBYEAR , 2019) OR LIMIT-TO (PUBYEAR , 2018) OR LIMIT-TO (PUBYEAR , 2017) OR LIMIT-TO (PUBYEAR , 2016) OR LIMIT-TO (PUBYEAR , 2015) OR LIMIT-TO (PUBYEAR , 2014) OR LIMIT-TO (PUBYEAR , 2013) OR LIMIT-TO (PUBYEAR , 2012) OR LIMIT-TO (PUBYEAR , 2011) OR LIMIT-TO (PUBYEAR , 2010) OR LIMIT-TO (PUBYEAR , 2009) OR LIMIT-TO (PUBYEAR , 2008) OR LIMIT-TO (PUBYEAR , 2007) OR LIMIT-TO (PUBYEAR , 2006) OR LIMIT-TO (PUBYEAR , 2005) OR LIMIT-TO (PUBYEAR , 2004) OR LIMIT-TO (PUBYEAR , 2003) OR LIMIT-TO (PUBYEAR , 2002) OR LIMIT-TO (PUBYEAR , 2001)) AND (LIMIT-TO (DOCTYPE , "ar") OR LIMIT-TO (DOCTYPE , "re"))

<u>Embase (439 hits)</u>

Specifications:

- 2000 – 2023
- Articles and reviews
- Humans

Search strategy:

- ('overweight' AND 'epidemiology' AND 'micronutrient*' AND 'deficiency' OR (('double burden' OR 'dual burden' OR 'triple burden' OR 'coexistence' OR 'co-existence') AND nutrition*) OR (('double burden' OR 'dual burden' OR 'triple burden' OR 'coexistence' OR 'co-existence') AND (malnutrition OR undernutrition)) OR (('overweight' AND 'epidemiology' AND 'micronutrients' AND 'deficiency' OR (('double burden' OR 'dual burden' OR 'triple burden') AND nutrition*) OR ((overweight OR obesity OR obese OR overnutrition) AND (micronutrient* OR anemia* OR stunt* OR underweight OR 'short stature' OR 'short height' OR undernutrition OR undernourished))) AND ('obesity/prevention and control' AND 'humans' OR ((normal AND body AND weight OR 'body mass index') AND ('diet' OR 'exercise') NOT 'weight gain/drug effects')))) AND ('latin america*' OR 'hispanic or latino' OR latin* OR

'antigua and barbuda*' OR 'argentin*' OR 'aruba*' OR 'baham*' OR 'barbad*' OR 'belize*' OR 'bolivia*' OR 'brazil*' OR 'british virgin island*' OR 'west indies' OR 'cayman island*' OR 'chile*' OR 'colombia*' OR 'costa rica*' OR 'cuba*' OR 'curacao' OR 'curaçao' OR 'dominica*' OR 'dominican republic' OR 'ecuador*' OR 'el salvador' OR 'salvadoran*' OR 'grenad*' OR 'guatemala*' OR 'guyan*' OR 'haiti*' OR 'hondura*' OR 'jamaica*' OR 'mexic*' OR 'nicaragua*' OR 'panama*' OR 'paraguay*' OR 'peru*' OR 'puerto rico' OR 'puerto rica*' OR 'sint maarten' OR 'st. maarten' OR 'saint martin' OR 'st. martin' OR 'saint kitts and nevis' OR 'st. kitts and nevis' OR 'saint lucia' OR 'st. lucia' OR 'saint vincent and the grenadines' OR 'st. vincent and the grenadines' OR 'suriname*' OR 'trinidad and tobago' OR 'turks and caicos islands' OR 'uruguay*' OR 'venezuela*' OR 'united states virgin island*') AND ([article]/lim OR [review]/lim) AND [humans]/lim AND [2000-2023]/py

1.3 Exclusion criteria

The exclusion criteria entailed the following:

- Design: Theoretical or simulation (modeling) designs, laboratory experiments or intervention studies. Duplicate publications from the same study will also be included in the initial screening for further assessment of the full text.
- Setting: Studies conducted in regions other than Latin America and the Caribbean based on the World Bank regions classification.[1]
- Population: Individuals with pre-existent chronic conditions (e.g. cancer, chronic kidney disease chronic liver disease, type 2 diabetes or other non-communicable diseases).
- Outcome: DBM at the population level and DBM measured subjectively or by self-reported data (micronutrient deficiencies measured by intake, self-reported BMI).
- Metric: Studies where a prevalence estimate cannot be extrapolated.

1.4 Extracted data

In Covidence, the following information was extracted:

- General details: author, year of publication
- Study design: cross-sectional, cohort, case-control
- Type of representativeness of the data: if nationally representative
- Data source: classified as Demographic Health Survey (DHS), a combination of DHS and other sources, National Nutrition Survey, or other.
- LAC sub-regions: Mesoamerica including Mexico and Central America, South America, or the Caribbean.
- Country
- Country income based on the World Bank income classification in 2021.[2]
- Area type: urban, semi-urban/semi-rural, rural, both, or unspecified.
- Gender of population: male, female, or both.
- Age group of population: pre-school 0-5 years, school-age 5-12y, adolescents 12-18y, adults 18-60, elderly >60y, multiple age groups.

In the Excel template, the following DBM specifications were extracted:

- Level of assessment: individual, household/pair, and across the life course
- Definition of DBM: ‘child (<18 years) with undernutrition + overnutrition’, ‘adult (>18 years) with undernutrition + overnutrition’, ‘child with undernutrition + adult with overnutrition’, and ‘child with overnutrition + adult with undernutrition’.
- Typology of DBM derived by the type of undernutrition (stunting, wasting, underweight, anemia, other micronutrient deficiencies and mixed referring to the combination of various types of undernutrition) and overnutrition (overweight) among those below 18 years, and type of undernutrition (underweight, short stature, anemia, other micronutrient deficiencies) and overnutrition (overweight + central obesity) among individuals above 18 years.
- Indicator used to characterize the type of undernutrition (e.g. heigh-for-age to measure stunting) and overnutrition (e.g. BMI to measure overweight).
- Prevalence estimate for DBM as the number of cases and total population, if % were reported, calculations were carried out to obtain the required figures.

Table S1. List of assumptions of eligible studies

Author, year	Assumption/note
Barquera 2007[3]	None.
Barreto 2003[4]	Assumed age is 65 years old.
Bassete 2014[5]	None.
Bernabé-Ortiz 2022[6]	The age of mothers and children was estimated at each follow-up point based on baseline age + years of follow-up.
Caleyachetty 2018[7]	Cases are calculated from the total sample size and prevalence % provided in the forest plot.
Conde 2014[8]	The year of data collection could not be estimated.
Costa 2013[9]	None.
De Menezes Toledo Florêncio 2001[10]	None.
Dieffenbach 2012[11]	None.

Doak 2005[12]	The study was included only in the systematic literature review, not in the meta-analysis as the estimate could not be categorized into any of the DBM definitions. Unable to determine specific indicators to measure overnutrition and undernutrition.
Doak 2016[13]	None.
Eckhardt 2008[14]	The study was only included in the systematic literature review. DBM estimated could not be extracted.
Felix-Beltran 2020[15]	None.
Ferreira 2017[16]	None.
Fooken 2022[17]	Cases calculated with data from Appendix Table A. Observed data extracted.
Freire 2014[18]	None.
Garrett 2005[19]	None.
Géa-Horta 2016[20]	None.
Ghattas 2020[21]	Observed data extracted.
Gubert 2017[22]	Number of cases used for the prevalence, not the %.
Jardim-Botelho 2016[23]	Overnutrition is considered the risk of obesity [weight for length z score: > 1 SD]
Jones 2017[24]	The prevalence of anemia + abdominal obesity for adult women could not be extracted as it is not presented in the paper.
Jones 2017[25]	Total populations were extracted from Figure 1 1 and prevalence % of DBM typologies from the 4th paragraph in the results section.
Jones-Smith 2007 [26]	DBM measured at the life course level. The study was included only in the systematic literature review, as not enough evidence was found to conduct meta-analyzes of DBM estimates at the life course level.
Kroker-Lobos 2014[27]	None.
Lee 2010[28]	None.
Lee 2012[29]	None.
Lee 2017[30]	None.
Lee 2021[31]	None.
Lerm 2021[32]	None.
Leroy 2014[33]	None.
Lourenço 2015[34]	is DBM measured at the life course level. The study was included only in the systematic literature review, as not enough evidence was found to conduct meta-analyzes of DBM estimates at the life course level.
Mendoza-Quispe 2021[35]	None.
Oliveira 2023[36]	Assumed the setting is urban.
Otten 2022[37]	The DBM estimate derived from 'mothers with anemia + child with overweight' was not extracted as the indicator of weight for age (z score) was used to determine overweight in children.
Oviedo-Solis 2022[38]	None.
Pajuelo Ramírez 2016[39]	Assumed the following number of the total population:<br>- Overweight + stunting = 3764<br>- Overweight + anemia = 2808<br>- Overweight + vitamin A deficiency = 1524
Palma Gutierrez 2019[40]	None.
Parra 2015[41]	Only extracted the following DBM combinations:<br>- Mother with overweight + at least one child with stunting, the rest normal<br>- Mother with underweight + at least one child with overweight, the rest normal
Parra 2018[42]	None.
Parra 2018[43]	None.
Pomati 2021[44]	None.
Popkin 2020[45]	None.
Ramirez-Zea 2014[46]	None.
Raphaël 2005[47]	None.
Ribeiro-Silva 2021[48]	Assumed the population is rural + urban.
Rivas 2018[49]	Cases calculated with data from Appendix Table A. Observed data extracted.
Rivas-Marino 2015[50]	None.
Rodríguez Ramos 2013[51]	None.
Rodríguez-Zúñiga 2015[52]	None.
Samper-Ternent 2012[53]	None.
Sanson-Rosas 2021[54]	The age of mothers assumed 15-49 years.
Sarmiento 2014[55]	None.
Sawaya 2004[56]	None.
Severi 2014[57]	The year of data collection was estimated. Assumed that in children (6 and 11 years old), overweight was estimated using BMI-for-age.
Syed 2016[58]	None.
Temponi 2020[59]	None.
Uzêda 2019[60]	Assumed age range from 13-18 years.
Varela-Silva 2012[61]	None.
Williams 2020[62]	The study was included only in the systematic literature review, not in the meta-analysis as they provided DBM estimates could not be extracted.

Table S2. DBM typologies identified and included

			Undernutrition									
			Children 0-18 years						Adults > 18 years			
			Stunting	Wasting	Under-weight	Anemia	Other micro-nutrient deficiencies~	Mixed under-nutrition **	Short stature	Under-weight	Anemia	Other micro-nutrient deficiencies++
**Over-nutrition**	**Children 0-18 years**	Overweight	X	-	-	X	X	X	-	X	-	-
	**Adults >18 years**	Overweight	X	X	X	X	X	X	X	-	X	X
		Central obesity	X	-	-	-	-	-	X	X	-	-

+ Other micronutrient deficiencies in children included: zinc deficiency, selenium deficiency, copper deficiency, and vitamin A deficiency. ++ Other micronutrient deficiencies in adults included: zinc deficiency, vitamin A deficiency, vitamin B12 deficiency, and folate deficiency. ~ Mixed indicates the following combination of types of undernutrition: stunting + underweight, stunting + anemia, wasting + underweight, wasting + stunting + underweight. For children, the methods used to collect and diagnose malnutrition outcomes were: overweight (weight-for-height or body mass index-for-age), stunting (height-for-age), wasting (weight-for-age or weight-for-height), underweight (weight-for-age or body mass index-for-age), anemia (laboratory cut-off by each study), other micronutrient deficiencies (laboratory cut-off by each study), mixed undernutrition (combination of the methods mentioned above). For adults, the methods used to collect and diagnose malnutrition outcomes were: overweight (body mass index), central obesity (waist circumference, waist-to-hip ratio, waist-to-height ratio), short stature (height cut-off specific by each study), underweight (body mass index), anemia (laboratory cut-off by each study), other micronutrient deficiencies (laboratory cut-off by each study).

Section 2: Results

Table S3. Evidence table of eligible studies

Author, year	Countries	Type of area	Study design	Year of data collection	Data source	DBM Level	DBM typologies extracted~	Risk of bias
Barquera 2007[3]	MEX	Urban and rural	Cross-sectional	1998 - 1999	Nutrition survey	Household / pair	10 ; 15	Low
Barreto 2003[4]	BRA	Unspecified	Cross-sectional from a cohort data point	1996	Other	Individual	7	Moderate
Bassete 2014[5]	ARG	Unspecified	Cross-sectional	2005	Nutrition survey	Household / pair	10	Moderate
Bernabé-Ortiz 2022[6]	PER	Urban and rural	Cohort	2002 - 2017	Other	Household / pair	10	Low
Caleyachetty 2018[7]	ARG ; BLZ ; BOL ; VGB ; CHL ; CRI ; DMA ; GTM ; GUY ; HND ; JAM ; PER ; KNA ; SUR ; URY	Urban and rural	Cross-sectional	2004 - 2012	Other	Individual	1	Low
Conde 2014[8]	BRA	Urban and rural	Cross-sectional	1974 - 2009	DHS + other	Household / pair ; Individual	1 ; 2 ; 7 ; 10	Moderate
Costa 2013[9]	BRA	Urban and rural	Cross-sectional	2006	Nutrition survey	Individual	7 ; 8	Low
De Menezes Toledo Florêncio 2001[10]	BRA	Urban and rural	Cross-sectional	1999	Other	Individual	1 ; 5	Moderate
Dieffenbach 2012[11]	BOL ; BRA ; COL ; DOM ; GTM ; HTI ; HND ; NIC ; PER	Urban and rural	Cross-sectional	1991 - 2008	DHS only	Household / pair	10	Low
Doak 2005[12]	BRA	Urban and rural	Cross-sectional	1989	Nutrition survey	Household / pair	No typologies extracted.	Low
Doak 2016[13]	GTM	Urban; Semi-urban	Cross-sectional	2011	Other	Household / pair ; Individual	5 ; 10	Moderate
Eckhardt 2008[14]	MEX ; PER	Urban and rural	Cross-sectional	1998 - 2000	DHS + other	Individual	No typologies extracted.	Low
Felix-Beltran 2020[15]	MEX	Urban and rural	Cross-sectional	2011-2012	Nutrition survey	Household / pair	10	Low
Ferreira 2017[16]	BRA	Unspecified	Cross-sectional	2008	Other	Individual	5 ; 6	Moderate
Fooken 2022[17]	BOL ; BRA ; COL ; DOM ; GTM ; HND ; HTI ; NIC ; PER	Urban and rural	Cross-sectional	1991 - 2017	DHS only	Household / pair	10	Low
Freire 2014[18]	ECU	Urban and rural	Cross-sectional	2012	DHS + other	Household / pair ; Individual	1 ; 2 ; 3 ; 7 ; 8 ; 10 ; 13 ; 14	Low
Garrett 2005[19]	BOL ; BRA ; COL ; DOM ; GTM ; HTI ; NIC ; PER	Urban and rural	Cross-sectional	1991 - 1998	DHS only	Household / pair	10	Low
Géa-Horta 2016[20]	BRA	Urban and rural	Cross-sectional	2006 - 2007	DHS	Household / pair	10	Low
Ghattas 2020[21]	BRB ; BLZ ; COL ; DOM ; SLV ; GTM ; GUY ; HTI ; HND ; MEX ; PRY ; PER ; SUR	Urban and rural	Cross-sectional	2010 - 2016	DHS + other	Individual	1	Low
Gubert 2017[22]	BRA	Urban and rural	Cross-sectional	2006	DHS only	Household / pair	10	Low

Jardim-Botelho 2016[23]	BRA	Urban	Cross-sectional	2009 - 2010	Other	Individual	2 ; 3	Moderate
Jones 2017[24]	MEX	Urban and rural	Cross-sectional	2012	Nutrition survey	Individual	2 ; 7	Low
Jones 2017[25]	BOL	Urban and rural	Cross-sectional from a cohort data point	2015	Other	Household / pair ; Individual	1 ; 2 ; 7 ; 10 ; 13	Low
Jones-Smith 2007[26]	MEX	Semi-urban	Cohort	2000 - 2005	Other	Across the life course	No typologies extracted.	Moderate
Kroker-Lobos 2014[27]	MEX	Urban and rural	Cross-sectional	2012	Nutrition survey	Household / pair ; Individual	1 ; 2 ; 7 ; 10	Low
Lee 2010[28]	GTM	Urban and rural	Cross-sectional	2000	Other	Household / pair	10	Low
Lee 2012[29]	GTM	Urban and rural	Cross-sectional	2000	Other	Household / pair	10	Low
Lee 2017[30]	GTM	Urban and rural	Cross-sectional	2000	Other	Household / pair	10	Low
Lee 2021[31]	ECU	Rural	Cross-sectional	2003 - 2013	Other	Household / pair	10 ; 13	Moderate
Lerm 2021[32]	HTI ; BOL ; SLV ; GTM ; HND ; PRY ; BLZ ; COL ; CUB ; DOM ; GUY ; MEX ; PER ; LCA ; SUR	Urban and rural	Cross-sectional	2008 - 2016	DHS + other	Individual	1	Low
Leroy 2014[33]	MEX	Rural	Cross-sectional	2003 - 2004	Other	Household / pair	10	Low
Lourenço 2015[34]	BRA	Urban	Cohort	2003 - 2009	Other	Household / pair ; Across the life course	No typologies extracted.	Moderate
Mendoza-Quispe 2021[35]	PER	Urban and rural	Cross-sectional	2009 - 2016	DHS only	Household / pair	16	Low
Oliveira 2023[36]	BRA	Urban	Cross-sectional	2007	Other	Individual	1 ; 2 ; 4	Moderate
Otten 2022[37]	BOL ; COL ; HTI ; HND ; DOM ; GUY ; GTM ; PER	Urban and rural	Cross-sectional	2001 - 2017	DHS only	Household / pair	10 ; 11 ; 13	Low
Oviedo-Solis 2022[38]	MEX	Urban and rural	Cross-sectional	2006 ; 2016	Nutrition survey	Individual	2	Low
Pajuelo Ramírez 2016[39]	PER	Urban and rural	Cross-sectional	2008 - 2010	Nutrition survey	Individual	1 ; 2 ; 3	Low
Palma Gutierrez 2019[40]	PER	Urban and rural	Cross-sectional	2017	DHS only	Individual	2	Low
Parra 2015[41]	COL	Urban and rural	Cross-sectional	2000 ; 2005 ; 2010	DHS only	Household / pair	10 ; 17	Low
Parra 2018[42]	COL	Urban and rural	Cross-sectional	2000 ; 2005 ; 2010	DHS only	Household / pair	10	Low
Parra 2018[43]	COL	Urban and rural	Cross-sectional	2005	DHS only	Household / pair	10	Low
Pomati 2021[44]	PER	Urban and rural	Cross-sectional	1996 ; 2000 ; 2008 ; 2010 ; 2016	DHS only	Household / pair	10 ; 11 ; 12 ; 16	Low
Popkin 2020[45]	BOL ; BRA ; COL ; DOM ; GTM ; GUY ; HTI ; HND ; MEX ; NIC ; PER	Urban and rural	Cross-sectional	1988 - 2017	DHS only	Household / pair	1 ; 10 ; 11 ; 17	Low
Ramirez-Zea 2014[46]	GTM	Urban and rural	Cross-sectional	2008	DHS only	Household / pair ; Individual	1 ; 2 ; 5 ; 7 ; 10	Low
Raphaël 2005[47]	HTI	Urban	Cross-sectional	2003	Other	Household / pair	16	Moderate
Ribeiro-Silva 2021[48]	BRA	Urban	Cross-sectional	2009 - 2017	Other	Individual	1	Moderate
Rivas 2018[49]	ARG	Urban	Cross-sectional	2014	Other	Individual	2 ; 7	Moderate
Rivas-Marino 2015[50]	MEX	Urban	Cross-sectional	2009 - 2010	Other	Individual	9	Low

Rodríguez Ramos 2013[51]	MEX	Urban	Cross-sectional	2011 - 2012	Other	Individual	1	Moderate
Rodríguez-Zúñiga 2015[52]	PER	Urban	Cross-sectional	2014	Other	Individual	2	Moderate
Samper-Ternent 2012[53]	MEX	Urban	Cross-sectional	2006	Nutrition survey	Individual	7	Low
Sanson-Rosas 2021[54]	COL	Urban	Cross-sectional	2015	Nutrition survey	Household / pair	10	Low
Sarmiento 2014[55]	COL	Urban	Cross-sectional	2010	DHS + other	Household / pair ; Individual	1 ; 2 ; 7 ; 10	Low
Sawaya 2004[56]	BRA	Urban	Cross-sectional	1999	Other	Individual	1	Moderate
Severi 2014[57]	URY	Urban	Cross-sectional from a cohort data point	2004 - 2011	Other	Household / pair ; Individual	1	Low
Syed 2016[58]	MEX ; COL	Urban	Cross-sectional	2006 ; 200	Nutrition survey	Individual	2	Low
Temponi 2020[59]	BRA ; BOL ; COL ; PER	Urban	Cross-sectional	2006 - 2012	DHS only	Household / pair	10	Low
Uzêda 2019[60]	BRA	Urban	Cross-sectional	2009 ; 2015	Other	Individual	1	Low
Varela-Silva 2012[61]	MEX	Urban	Cross-sectional	2010	Other	Household / pair ; Individual	1 ; 5 ; 10	Moderate
Williams 2020[62]	MEX ; ECU ; COL	Urban	Cross-sectional	2010 ; 2006 ; 2012	Other	Individual	No typologies extracted.	Low

Abbreviations: Double burden of malnutrition (DBM); Demographic Health Survey (DHS). ~ The DBM typologies are the following are coded as follow: 1 “Individual level in children 0-18y - Overweight+stunting", 2 "Individual level in children 0-18y - Overweight+anemia", 3 "Individual level in children 0-18y - Overweight+ other micronutrient deficiencies", 4 " Individual level in children 0-18y - Overweight+mixed undernutrition", 5 " Individual level in adults >18y - Overweight+short stature", 6 " Individual level in adults >18y - Central obesity+short stature" 7 " Individual level in adults >18y - Overweight+anemia", 8 " Individual level in adults >18y - overweight+other nutronutrient deficiencies", 9 " Individual level in adults >18y - Central obesity+underweight", 10 "Household level – adult overweight+child stunting", 11 "Household level - adult overweight+child wasting", 12" Household level – adult overweight+child underweight", 13" Household level – adult overweight+child anemia", 14 "Household level – adult overweight+child other micronutrient deficiencies", 15 " Household level – adult central obesity+child stunting", 16 " Household level – adult overweight+child mixed undernutrition" and 17 " Household level - Child overweight+adult underweight".

Typology

Typology	PER	BRA	COL	MEX	GTM	BOL	HTI	DOM	HND	NIC	ECU	GUY	ARG	URY	BLZ	SUR	SLV	PRY	CRI	CHL	BRB	CUB	DMA	VGB	JAM	KNA	LCA	Total
Household (adult + child)<br>*Overweight + stunting*	26	8	20	8	14	14	12	9	6	8	3	2	1	1														**132**
Individual - 0-18y<br>*Overweight + stunting*	6	16	5	7	6	5	4	4	5	2	1	4	1	3	3	3	2	2	1	1	1	1	1	1	1	1	1	**88**
Household (adult + child)<br>*Overweight + wasting*	8	1	3	2	3	3	3	3	3	3		2																**34**
Household (Child + adult)<br>*Overweight + underweight*	2	1	5	2	2	2	2	2	2	2		1																**23**
Individual - 0-18y<br>*Overweight + anemia*	3	4	2	7	1	1					1		1															**20**
Household (adult + child)<br>*Overweight + mixed*	16						1																					**17**
Individual >18y<br>*Overweight + anemia*		4	1	3	1	1					1		1															**12**
Household (adult + child)<br>*Overweight + anemia*	1				1	2	1		1		3	1																**10**
Individual - 0-18y<br>*Overweight + micr def.*	1	3									1																	**5**
Individual >18y<br>*Overweight + short stature*		2		1	2																							**5**
Household (adult + child)<br>*Overweight + underweight*	5																											**5**
Individual >18y<br>*Central obesity + short stature*		2																										**2**
Individual >18y<br>*Overweight + micr def.*		1									1																	**2**
Household (adult + child)<br>*Central obesity + stunting*				2																								**2**
Individual - 0-18y<br>*Overweight + mixed*		1																										**1**
Individual >18y<br>*Central obesity + underweight*				1																								**1**
Household (adult + child)<br>*Overweight + micr. def*											1																	**1**
**Total**	**68**	**43**	**36**	**33**	**30**	**28**	**23**	**18**	**17**	**15**	**12**	**10**	**4**	**4**	**3**	**3**	**2**	**2**	**1**	**1**	**1**	**1**	**1**	**1**	**1**	**1**	**1**	**360**

Figure S1. Heat map of the frequency of DBM typologies by country

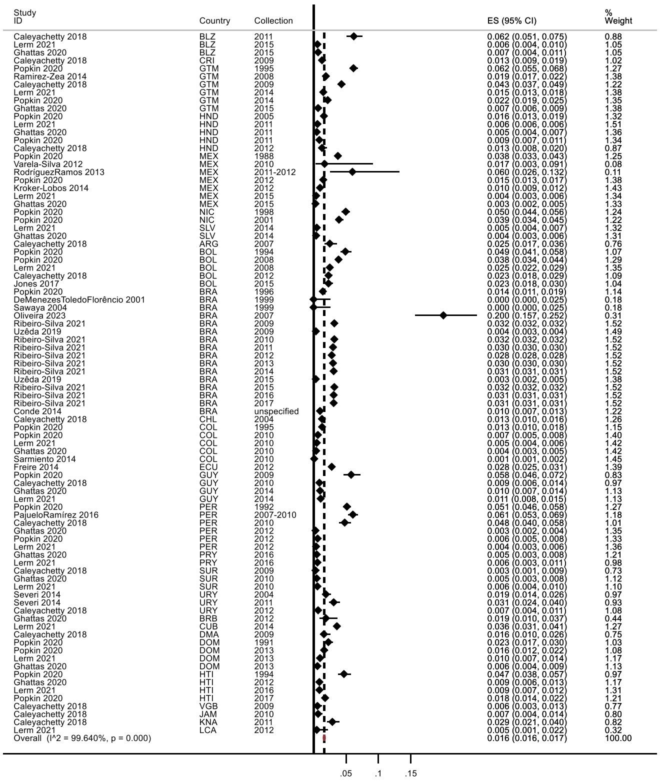

Figure S2. Forest plot – Individual level in children 0-18 years, typology overweight + stunting

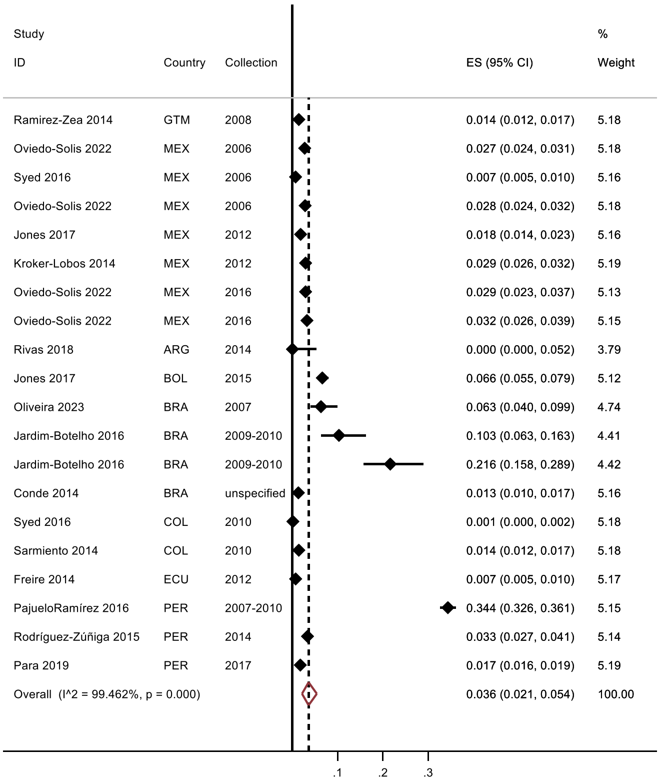

Figure S3. Forest plot – Individual level in children 0-18 years, typology overweight + anemia

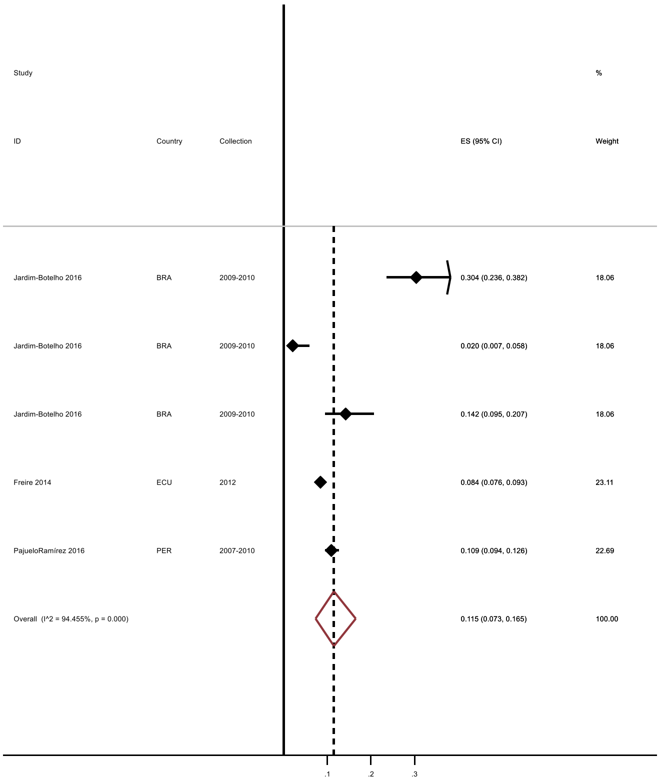

Figure S4. Forest plot – Individual level in children 0-18 years, typology overweight + other micronutrient deficiencies
Other micronutrient deficiencies include zinc deficiency (2), selenium deficiency (1), copper deficiency (1) and vitamin A deficiency (1).

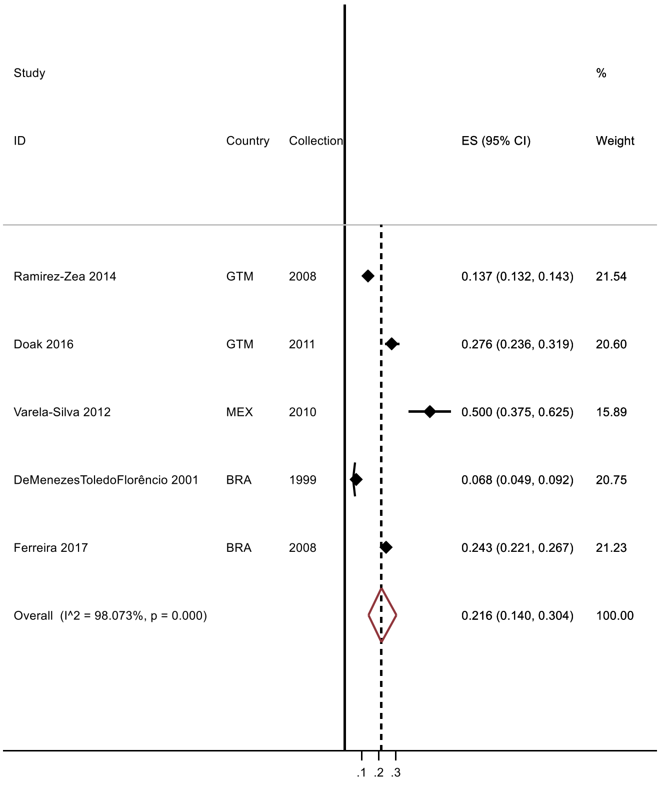

Figure S5. Forest plot – Individual level in adults >18 years, typology overweight + short stature

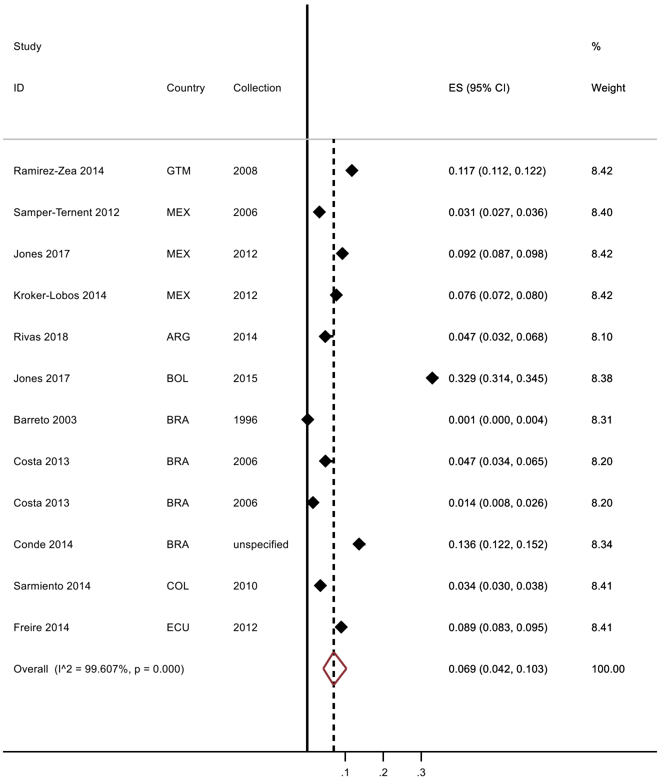

Figure S6. Forest plot – Individual level in adults >18 years, typology overweight + anemia

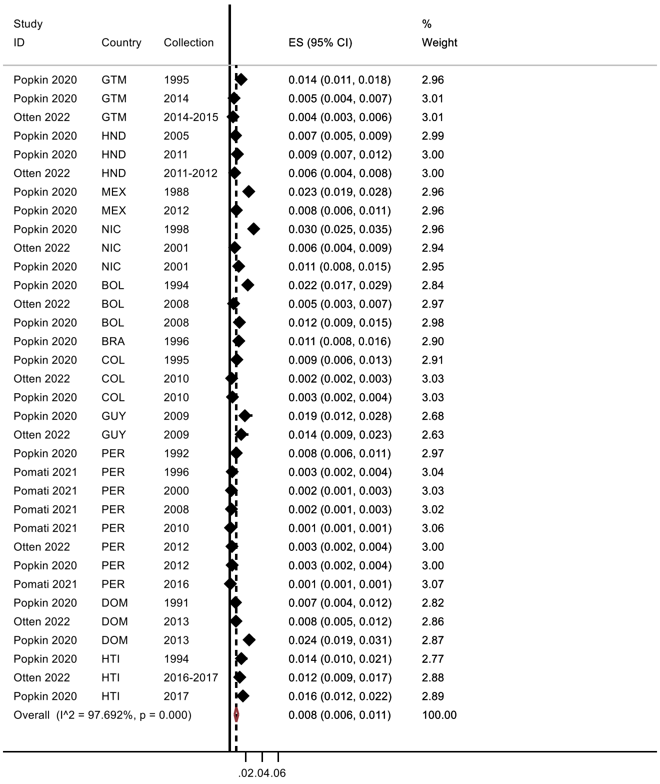

Figure S7. Forest plot – Household level, adult with overweight + child with wasting

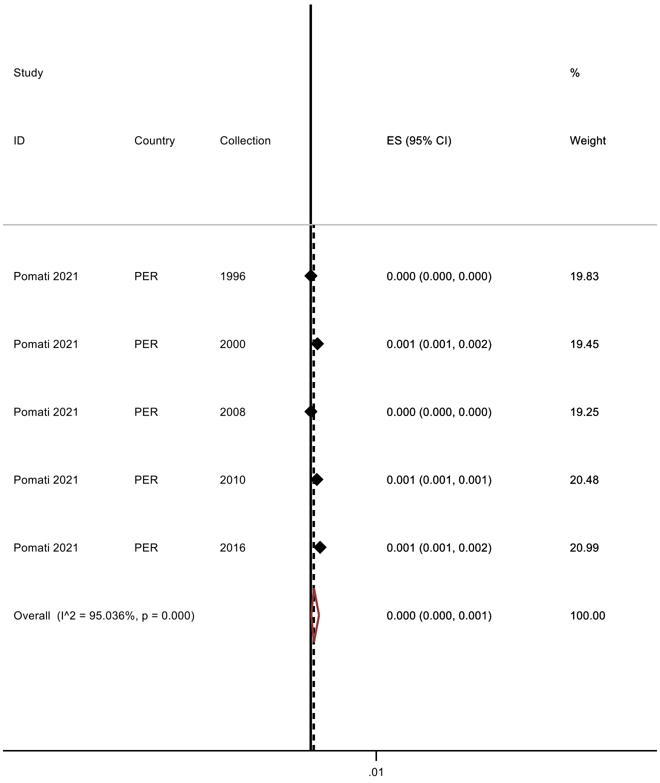

Figure S8. Forest plot – Household level, adult with overweight + child with underweight

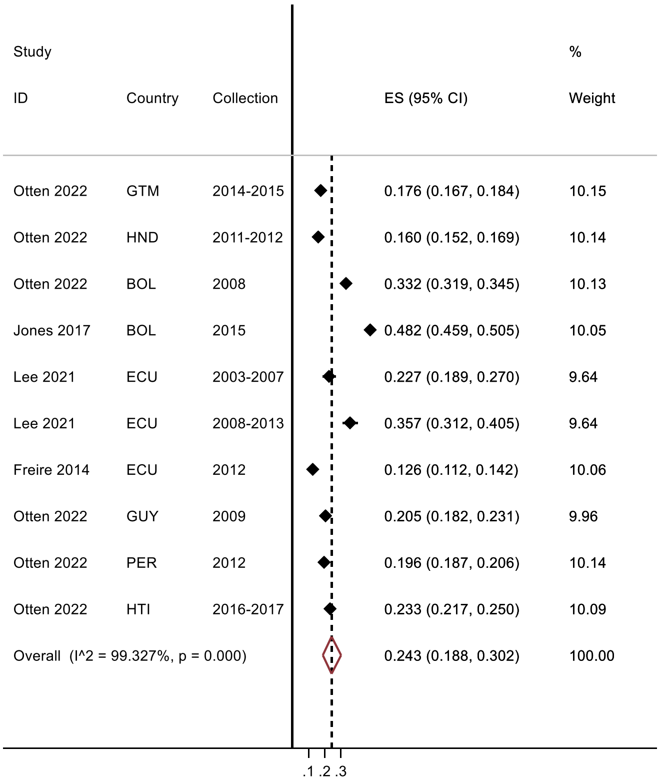

Figure S9. Forest plot – Household level, adult with overweight + child with anemia

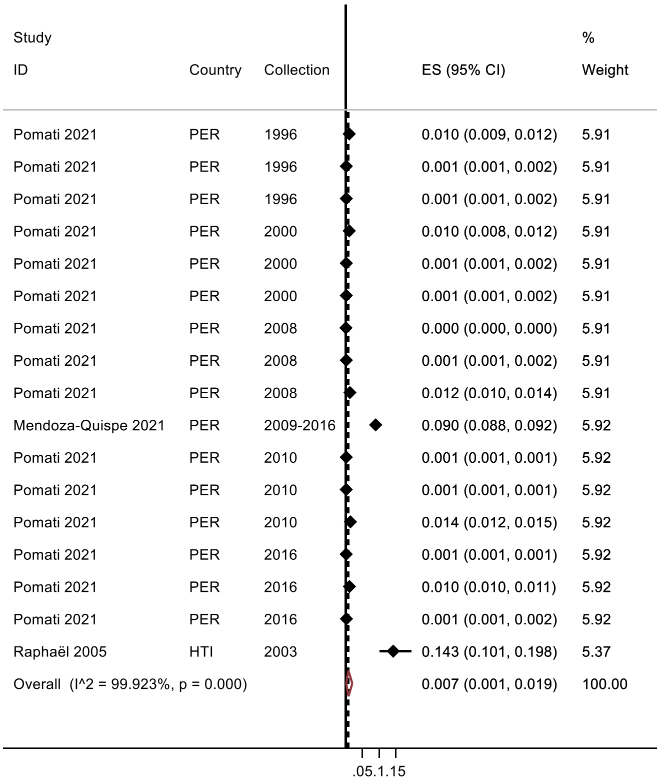

Figure S10. Forest plot – Household level, adult with overweight + child with mixed undernutrition
Mixed undernutrition indicates the following combination of types of undernutrition: stunting + underweight (5), stunted OR wasting (1), underweight + wasting (5), wasting + stunted + underweight (6).

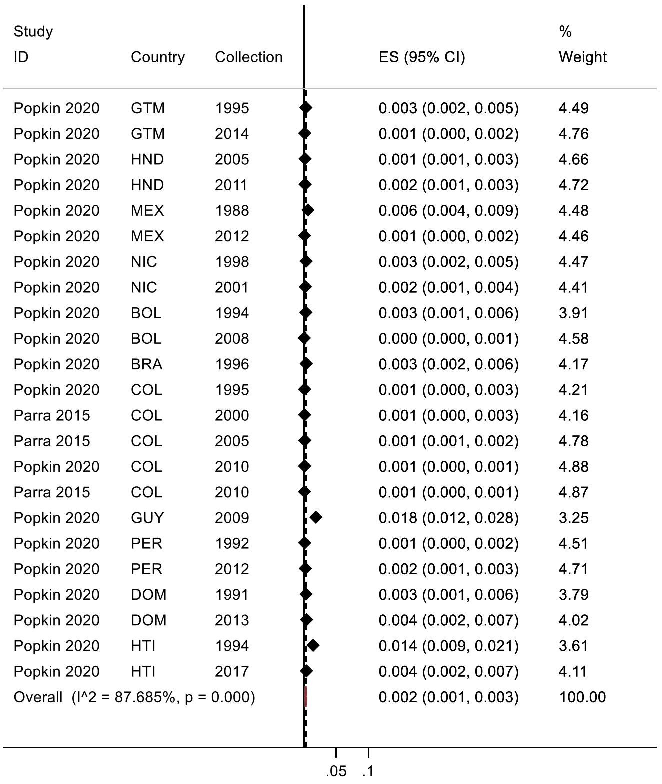

Figure S11. Forest plot – Household level, child with overweight + adult with underweight

Table S4. Univariate meta-regressions: Heterogeneity exploration for the outcomes related to DBM typologies at the individual level

			**Children 0-18y<br>Overweight + Stunting**		**Children 0-18y<br>Overweight + Anemia**		**Adult >18y<br>Overweight + Anemia**
		n	β (95% CI)	n	β (95% CI)	n	β (95% CI)
			p-value		p-value		p-value
**Region**							
	Mesoamerica	26	-0.005 (-0.013; 0.003)	8	-0.046 (-0.124; 0.032)	4	-0.012 (-0.135; 0.114)
			0.205		0.234		0.853
	South America	47**	0.022 (0.017; 0.026)	12**	0.069 (0.016; 0.121)	8**	0.090 (0.016; 0.163)
			<0.001		0.013		0.022
	Caribbean	15	-0.004 (-0.017; -0.008)	0	-	0	-
			0.502				
	Walt test (p value)*		0.407		-		-
**Country+**							
	Honduras	5	**-0.020 (-0.031; -0.008)**		-		-
			**0.001**				
	Mexico	7	**-0.015 (-0.028; -0.003)**		-		-
			**0.018**				
	El Salvador	2	**-0.023 (0.044; -0.002)**		-		-
			**0.029**				
	Colombia	5	**-0.023 (0.034; 0.012)**		-		-
			**<0.001**				
	Brazil	16**	0.027 (0.023; 0.032)		-	4**	0.053 (-0.017; 0.123))
			<0.001				0.110
	Bolivia		-		-	1	**0.276 (0.132; 0.419)**
							**0.004**
	Walt test (p value)*		0.066		0.671		0.053
**Income**							
	Low-middle-income	18	-0.002 (-0.011; 0.007)	1	0.018 (-0.161; 0.198)	1	**0.262 (0.166; 0.358)**
			0.594		0.831		**<0.001**
	Upper-middle-income	63**	0.021 (0.017; 0.025)	19**	0.048 (0.006; 0.089)	12**	0.067 (0.038; 0.096)
			<0.001		0.027		<0.001
	High-income	7	-0.004 (-0.024; 0.015)	0	-	0	-
			0.667				
	Walt test (p value)*		0.806		-		-
**Type of area**							
	Rural	0	-	1	-0.009 (-0.191; 0.172)	0	-
					0.916		
	Urban/semi-urban	4	-0.013 (-0.031; 0.004)	3	0.079 (-0.062; 0.220)	0	-
			0.130		0.253		
	Rural + urban	82**	0.021 (0.017; 0.024)	15**	0.042 (-0.002; 0.087)	10**	0.082 (0.020; 0.143)
			<0.001		0.061		0.014
	Unspecified	2	0.001 (-0.130; 0.133)	1	-0.042 (-0.350; 0.265)	2	0.054 (-0.158; 0.267)

			0.984		0.253		0.581
	Walt test (p value)*		0.315		0.6742		-
**Year of data collection**							
	1988 – 2000	11	**0.022 (0.008; 0.036)**	0	-	1	-0.128 (-0.350; 0.094)
			**0.002**				0.220
	2001 – 2011	36	0.002 (-0.006; 0.009)	10**	0.072 (0.014; 0.130)	5	-0.079 (-0.205; 0.048)
			0.660		0.017		0.190
	2012 – 2017	40**	0.018 (0.013; 0.023)	9	-0.045 (-0.128; 0.038)	5**	0.129 (0.040; 0.218)
			<0.001		0.271		0.010
	Unspecified	1	-0.008 (-0.045; 0.029)	1	-0.059 (-0.241; 0.121)	1	0.007 (-0.212; 0.227)
			0.677		0.499		0.941
	Walt test (p-value)*		**0.019**		0.489		0.399
**Number of participants**							
	≤ median	44**	0.021 (0.013; 0.028)	10**	0.089 (0.034; 0.145)	6**	0.100 (0.015; 0.186)
			<0.001		0.003		0.025
	>median	44	-0.001 (-0.009; 0.008)	10	-0.072 (-0.145; 0.001)	6	-0.027 (-0.144; 0.090)
			0.854		0.052		0.619
**Risk of bias**							
	Low risk	73**	0.012 (0.009; 0.015)	14**	0.045 (-0.001; 0.090)	9**	0.094 (0.026; 0.161)
			<0.001		0.054		0.011
	Moderate risk	15	**0.019 (0.015; 0.022)**	6	0.018 (-0.079; 0.115)	3	-0.031 (-0.170; 0.107)
			**<0.001**		0.702		0.626

** The reference, is the most frequent category; the outputs of each meta-regression presented on this table should be interpreted when compared to the reference. ~Median number of participants were as followed: Children 0-18y - Overweight + Stunting (4300), Children 0-18y - Overweight + Anemia (3723), Adult >18y - Overweight + Anemia (4526). + The meta-regression for countries presented in the table were the ones shown as statistically significant; all countries were included in the model (non-significant values not shown). Country reference: Children 0-18y - Overweight + Stunting (Brazil), Children 0-18y - Overweight + Anemia (Mexico), Adult >18y - Overweight + Anemia (Brazil). *Walt test was conducted for the univariate with 3 or more subgroup categories of dichotomous variables. The bold results indicate the significance of the univariate meta-regression based on an alpha of 0.05. Based on these results, multivariate meta-regression were performed (Results presented in Table S5).

Table S5. Univariate meta-regressions: Heterogeneity exploration for the outcomes related to DBM typologies at the household/pair level

			**Adult with overweight + child with stunting**		**Adult with overweight + child with wasting**		**Adult with overweight + child with anemia**		**Adult with overweight + child with mixed undernutrition**		**Child with overweight + adult underweight**
		n	β (95% CI)	n	β (95% CI)	n	β (95% CI)	n	β (95% CI)	n	β (95% CI)
			p-value		p-value		p-value		p-value		p-value
**Region**											
	Mesoamerica	36	**0.042 (0.025; 0.060)**	11	0.007 (-0.002; 0.017)	2	-0.107 (-0.318; 0.105)	-	-	8	0.001 (-0.012; 0.014)
			**<0.001**		0.118		0.272				0.908
	South America	75**	0.088 (0.078; 0.098)	17**	0.003 (-0.002; 0.007)	7**	0.274 (0.172; 0.377)	16**	0.010 (-0.002; 0.023)	11**	0.001 (-0.007; 0.10)
			<0.001		0.231		<0.001		0.107		0.744
	Caribbean	21	**-0.052 (-0.074; 0.030)**	6	0.011 (-0.007; 0.029)	1	-0.041 (-0.325; 0.243)	1	0.133 (-0.025; 0.290)	4	0.004 (-0.020; 0.028)
			**<0.001**		0.222		0.741		0.092		0.727
	Walt test (p-value)*				0.175		0.519		-		0.939
**Country+**											
	Honduras		-		-		-		-		-
	Mexico	8	**-0.034 (-0.057; -0.011)**		-		-		-		-
			**0.004**								
	El Salvador		-		-		-		-		-
	Colombia	20	**-0.045 (-0.060; -0.029)**		-		-		-		-
			**<0.001**								
	Brazil	8	**-0.075 (-0.098; -0.052)**		-		-		-		-
			**<0.001**								
	Guatemala	14	**0.081 (0.063; 0.100)**		-		-		-		-
			**<0.001**								
	Bolivia	14	**0.018 (0.000; 0.036)**		-		-		-		-
			**0.047**								
	Dominican Republic	9	**-0.072 (-0.095; -0.048)**		-		-		-		-
			**<0.001**								
	Haiti	12	**-0.067 (-0.088; -0.046)**		-		-	1	0.133 (-0.025; 0.290)		-
			**<0.001**						0.092		
	Peru	26**	0.105 (0.095; 0.116)		-		-	16**	0.010 (-0.002; 0.023)		-
			<0.001						0.107		
	Walt test (p value)*		**<0.001**		0.757		0.564		-		0.999
**Income**											
	Low-middle-income	40	0.020 (-0.018; 0.021)	12	0.007 (-0.002; 0.018)	4	0.090 (-0.064; 0.245)	1	0.133 (-0.025; 0.290)	8	0.001 (-0.013; 0.014)
			0.843		0.124		0.15		0.092		0.926
	Upper-middle-income	91**	0.092 (0.081; 0.102)	22**	0.004 (-0.001; 0.008)	6**	0.210 (0.110; 0.310)	16**	0.010 (-0.002; 0.023)	15**	0.002 (-0.006; 0.010)
			<0.001		0.108		0.001		0.107		0.628
	High-income	1	-0.029 (-0.138; 0.080)	0	-	0	-	0	-	0	-

			0.605								
	Walt test (p-value)*		0.852		-		-		-		-
**Type of area**											
	Rural	3	-0.0127 (-0.085; 0.060)	0	-	2	0.054 (-0.164; 0.271)	0	-	0	-
			0.731				0.586				
	Urban/semi-urban	2	0.074 (-0.047; 0.194)	0	-	0	-	1	0.133 (-0.025; 0.290)	0	-
			0.229						0.092		
	Rural + urban	127**	0.092 (0.083; 0.101)	34**	0.005 (0.001; 0.009)	8**	0.238 (0.147; 0.339)	16**	0.010 (-0.002; 0.023)	23	0.002 (-0.004; 0.008)
			<0.001		0.013				0.107		0.523
	Unspecified	0	-	0	-	0	-	0	-	0	-
	Walt test (p-value)*		0.454		-		-		-		-
**Year of data collection**											
	1988 – 2000	55**	0.091 (0.078; 0.105)	11	0.005 (-0.005; 0.016)	0	-	6	-0.016 (-0.046; 0.014)	10**	0.003 (-0.008; 0.015)
			<0.001		0.319				0.275		0.574
	2001 – 2011	51	-0.001 (0.021; 0.018)	12**	0.004 (-0.002; 0.011)	4	0.053 (-0.117; 0.222)	8**	0.020 (-0.000; 0.040)	8	-0.002 (-0.016; 0.013)
			0.900		0.182		0.494		0.052		0.793
	2012 – 2017	25	0.009 (-0.016; 0.033)	11	-0.001 (-0.010; 0.008)	6**	0.228 (0.124; 0.332)	3	-0.016 (-0.051; 0.020)	5	-0.001 (-0.19; 0.016)
			0.485		0.804		0.001		0.365		0.864
	Unspecified	1	-0.065 (-0.166; 0.036)	0	-	0	-	0	-	0	-
			0.203								
	Walt test (p-value)*		0.497		0.445		-		0.470		-
**Number of participants**											
	≤ median	66**	0.079 (0.066; 0.092)	17**	0.015 (0.006; 0.024)	5**	0.279 (0.161; 0.397)	10**	0.006 (-0.012; 0.024)	12**	0.003 (-0.008; 0.015)
			<0.001		0.002		<0.001		0.501		0.550
	>median	66	**0.025 (0.007; 0.042)**	17	**-0.012 (-0.022; -0.002)**	5	-0.060 (-0.223; 0.103)	7	0.011 (-.015; 0.038)	11	-0.002 (-0.015; 0.012)
			**0.006**		**0.022**		0.421		0.370		0.764
**Risk of bias**											
	Low risk	126**	0.092 (0.083; 0.101)	34**	0.005 (0.001; 0.009)	8**	0.238 (0.147; 0.330)	16**	0.010 (-0.002; 0.023)	23**	0.002 (-0.004; 0.008)
			<0.001		0.013		<0.001		0.107		0.523
	Moderate risk	6	-0.012 (-0.071; 0.046)	0	-	2	0.054 (-0.164; 0.271)	1	0.133 (-0.025; 0.290)	0	-
			0.674				0.586		0.092		

**The reference, is the most frequent category; the outputs of each meta-regression presented on this table should be interpreted when compared to the reference. ~Median number of participants were as followed: Adult with overweight + child with stunting (3967), Adult with overweight + child with wasting (4701), Adult with overweight + child with anemia (2219), Adult with overweight + child with mixed undernutrition (14812), Child with overweight + adult underweight (4486). + The meta-regression for countries presented in the table were the ones shown as statistically significant; all countries were included in the model (non-significant values not shown). Country reference: Adult with overweight + child with stunting (Peru), Adult with overweight + child with wasting (Peru), Adult with overweight + child with anemia (Ecuador), Adult with overweight + child with mixed undernutrition (Peru), Child with overweight + adult underweight (Colombia). The bold results indicate the significance of the univariate meta-regression based on an alpha of 0.05. Based on these results, multivariate meta-regression were performed (Results presented in Table S5).

Table S6. Multivariate meta-regressions: Heterogeneity exploration for the outcomes related to DBM typologies at the individual and household/pair level

			**Children 0-18y - Overweight + stunting**		**Adult >18y Overweight + Anemia**		**Adult overweight + child with stunting**
		n	β (95% CI)	n	β (95% CI)	n	β (95% CI)
			p-value		p-value		p-value
**Intercept**			0.015 (0.011; 0.018)		0.067 (0.038; 0.096)		0.103 (0.089; 0.116)
**Region**					-		
	Mesoamerica		-			36	0.004 (-0.013; 0.021)
					-		0.657
	Caribbean		-			21	**-0.069 (-0.094; -0.044)**
					-		**<0.001**
**Country+**							
	Honduras	5	-0.008 (-0.014; -0.002)		-		-
			0.009				
	Mexico	7	-0.006 (-0.015; 0.004)		-	8	**-0.037 (-0.062; -0.012)**
			0.272				**0.004**
	El Salvador	2	-0.10 (-0.027; 0.007)		-		-
			0.253		-		
	Colombia	5	-0.011 (-0.020; -0.003)			20	**-0.043 (-0.059; -0.028)**
			0.008		-		**<0.001**
	Brazil		-			8	**-0.073 (-0.097; -0.050)**
					-		**<0.001**
	Guatemala		-			14	**0.079 (0.058; 0.100)**
					-		**<0.001**
	Bolivia		-			14	0.019 (0.002; 0.037)
				1	**0.262 (0.166; 0.358)**		0.033
	Dominican Republic		-		**<0.001**	9	Dropped due to collinearity
	Haiti		-			12	0.004 (-0.024; 0.032)
**Income**							0.755
	Low-middle-income			1	Dropped due to collinearity		
**Year of data collection**							
	1988 – 2000	11	**0.028 (0.017; 0.039)**				-
			**<0.001**				
**Number of participants**							
	>median		-			66	0.002 (-0.010; 0.014)
							0.771
**Risk of bias**							
	Moderate risk	15	**0.016 (-0.012; 0.020)**				
			**<0.001**				

The bold results indicate the significance of the multivariate meta-regression based on Bonferroni's adjusted p-value of 0.007, to correct for multiple comparisons.

Table S7. The pooled period prevalence of DBM typologies stratified by the encountered source of heterogeneity covering 1998-2017

		Number of estimates	Pooled period prevalence (95%CI)~	Pooled period prevalence, %	I^2, %	Heterogeneity p-value
**Children 0-18y - Overweight + stunting**						
Pooled prevalence		88	0.016 (0.016 – 0.017)	1.6 (1.6 – 1.7)	99.6	**<0.001**
Stratified by year of data collection	1988 – 2000	11	0.029 (0.019 – 0.041)	2.3 (1.9 – 10.3)	99.8	**<0.001**
	2001 – 2011	36	0.019 (0.016 – 0.021)	1.9 (1.6 – 2.1)	99.4	**<0.001**
	2012 – 2017	40	0.013 (0.013 – 0.014)	1.3 (1.3 – 1.4)	96.9	**<0.001**
	Unspecified	1	NA	NA	NA	**NA**
Stratified by risk of bias	Low risk of bias	73	0.015 (0.013 – 0.018)	1.5 (1.3 – 1.8)	98.7	**<0.001**
	Moderate risk of bias	15	0.029 (0.028 – 0.030)	2.3 (2.8 – 3.0)	99.0	**<0.001**
**Adult >18y - Overweight + Anemia**						
Pooled prevalence		12	0.069 (0.042 – 0.103)	6.9 (4.2 – 10.3)	99.6	**<0.001**
Stratified by country	Brazil	4	0.035 (0.000 – 0.124)	2.5 (0.0 – 12.4)	99.3	**<0.001**
	Colombia	1	NA	NA	NA	NA
	Ecuador	1	NA	NA	NA	NA
	Mexico	3	NA	NA	NA	NA
	Bolivia	1	NA	NA	NA	NA
	Guatemala	1	NA	NA	NA	NA
	Argentina	1	NA	NA	NA	NA
**Adult with overweight + child with stunting**						
Pooled prevalence		132	0.085 (0.077 – 0.093)	8.5 (7.7 – 9.3)	99.3	**<0.001**
Stratified by region	Mesoamerica	36	0.126 (0.107 – 0.147)	12.6 (10.7 – 14.7)	99.1	**<0.001**
	South America	75	0.084 (0.076 – 0.093)	8.4 (7.6 – 9.3)	99.3	**<0.001**
	Caribbean	21	0.035 (0.030 – 0.041)	3.5 (3.0 – 4.1)	88.6	**<0.001**
Stratified by country	Brazil	8	0.030 (0.025 – 0.035)	3.0 (2.5 – 3.5)	78.5	**<0.001**
	Uruguay	1	NA	NA	NA	**NA**
	Colombia	20	0.061 (0.055 – 0.067)	6.1 (5.5 – 6.7)	95.5	**<0.001**
	Ecuador	3	NA	NA	NA	**NA**
	Mexico	8	0.074 (0.052 – 0.099)	7.4 (5.2 – 9.9)	98.3	**<0.001**
	Bolivia	14	0.120 (0.102 – 0.139)	12.0 (10.2 – 13.9)	98.1	**<0.001**
	Guatemala	14	0.183 (0.161 – 0.205)	18.3 (16.1 – 20.5)	97.9	**<0.001**
	Peru	26	0.106 (0.093 – 0.119)	10.6 (9.3 – 11.9)	99.1	**<0.001**
	Argentina	1	NA	NA	NA	**NA**
	Nicaragua	8	0.099 (0.078 – 0.122)	9.9 (7.8 – 12.2)	97.7	**<0.001**
	Haiti	12	0.036 (0.028 – 0.045)	3.6 (2.8 – 4.5)	91.6	**<0.001**
	Guyana	2	NA	NA	NA	**NA**
	Dominican Republic	9	0.033 (0.028 – 0.039)	3.3 (2.8 – 3.9)	79.0	**<0.001**
	Honduras	6	11.6 (0.097 – 0.135)	11.6 (9.7 – 13.5)	97.4	**<0.001**

Abbreviations: double burden of malnutrition (DBM); 95% confidence interval (95%CI) ~ Pooled period prevalence with corresponding 95%CI were obtained using a random effect meta-analysis of proportions (command metaprop). Freeman-Tukey double arcsine transformation was used to stabilize variances from extreme proportional estimates. Pooled period prevalence were only estimated for those with a minimum of 5 estimates. The bold results indicate the significance of the univariate meta-regression based on an alpha of 0.05.

Table S8. Stratified analysis – Pooled period prevalence of DBM typologies stratified by year of data collection

	Year of data collection	Number of estimates	Pooled period prevalence (95%CI)~	Pooled period prevalence, %	I^2, %
**DBM typologies at the individual level**					
Children 0-18y with overnutrition + undernutrition					
Overweight + stunting	1988 – 2000	11	0.029 (0.019 – 0.041)	2.9 (1.9 – 4.1)	96.9
	2001 – 2011	36	0.019 (0.016 – 0.021)	1.9 (1.6 – 2.1)	99.8
	2012 - 2017	40	0.013 (0.013 – 0.014)	1.3 (1.3 – 1.4)	99.4
	1988-2017	88	0.016 (0.016 – 0.017)	1.6 (1.6 – 1.7)	99.6
**DBM typologies at the household level**					
Adult with overnutrition + child with undernutrition					
Overweight + stunting	1988 – 2000	55	0.083 (0.070 – 0.096)	8.3 (7.0 – 9.6)	99.1
	2001 – 2011	51	0.086 (0.075 – 0.098)	8.6 (7.5 – 9.8)	99.2
	2012 - 2017	25	0.092 (0.072 – 0.115)	9.2 (7.2 – 11.5)	99.6
	1988-2017	132	0.085 (0.077 – 0.093)	8.5 (7.7 – 9.3)	99.3

Abbreviations: double burden of malnutrition (DBM); 95% confidence interval (95%CI) ~ Pooled period prevalence with corresponding 95%CI were obtained using a random effect meta-analysis of proportions (command metaprop). Freeman-Tukey double arcsine transformation was used to stabilize variances from extreme proportional estimates.

Table S9. Sensitivity analysis – Pooled period prevalences of DBM typologies limited to nationally representative data covering 1998-2017

	**Number of estimates**	**No. of participants, median [IQR]**	**Pooled prevalence (95%CI)~**	**Pooled prevalence, %**	**I^2, %**	**Heterogeneity p-value**
**DBM typologies at the individual level**						
Children 0-18y with overnutrition + undernutrition						
Overweight + stunting	82	4910 [2412 - 10165]	0.016 (0.016 – 0.017)	1.6 (1.6 – 1.7)	99.6	**<0.001**
Overweight + anemia	14	5439 [3660 - 8573]	0.027 (0.013 – 0.046)	2.7 (1.3 – 4.6)	99.6	**<0.001**
Overweight + micronutrient deficiencies	2	NA	NA	NA	NA	NA
Overweight + mixed⁺	0	NA	NA	NA	NA	NA
*Any DBM for this level**	98	4597 [2581 - 9164]	0.019 (0.018 – 0.020)	1.9 (1.8 – 2.0)	99.7	**<0.001**
Adults >18y – with overnutrition + undernutrition						
Overweight + short stature	1	NA	NA	NA	NA	NA
Central obesity + short stature	0	NA	NA	NA	NA	NA
Overweight + anemia	7	8014 [5605 - 15049]	0.078 (0.054 – 0.105)	7.8 (5.4 – 10.5)	99.3	**<0.001**
Overweight + micronutrient deficiencies	1	NA	NA	NA	NA	NA
Central obesity + underweight	1	NA	NA	NA	NA	NA
*Any DBM for this level**	10	7610 [5605 - 15049]	0.086 (0.049 – 0.132)	8.6 (4.9 – 13.2)	99.8	**<0.001**
**DBM typologies at the household/pair level**						
Adult with overnutrition + child with undernutrition						
Overweight + stunting	121	4299 [2350 - 7334]	0.083 (0.075 – 0.092)	8.3 (7.5 – 9.2)	99.3	**<0.001**
Overweight + wasting	34	4701 [2688 - 7970]	0.008 (0.006 – 0.011)	0.8 (0.6 – 1.1)	97.7	**<0.001**
Overweight + underweight	5	14812 [11565 - 26805]	0.000 (0.000 – 0.001)	0.0 (0.0 – 0.1)	95.0	**<0.001**
Overweight + anemia	7	5163 [1893 - 7220]	0.201 (0.156 – 0.250)	20.1 (15.6 – 25.0)	99.1	**<0.001**
Overweight + micronutrient deficiencies	1	NA	NA	NA	NA	NA
Central obesity + stunting	2	NA	NA	NA	NA	NA
Overweight + mixed⁺	16	14812 [11565 - 46199]	0.005 (0.000 – 0.015)	0.5 (0.0 – 1.5)	99.9	**<0.001**
*Any DBM for this level**	186	4784 [2545 - 8622]	0.054 (0.045 – 0.064)	5.4 (4.5 – 6.4)	99.8	**<0.001**
Child with overnutrition + adult with undernutrition						
Overweight + underweight	23	4486 [2688 - 7242]	0.002 (0.001 – 0.003)	0.2 (0.1 – 0.3)	87.7	<**0.001**

Abbreviations: double burden of malnutrition (DBM); 95% confidence interval (95%CI) ~Pooled period prevalence with corresponding 95%CI were obtained using a random effect meta-analysis of proportions (command metaprop). Freeman-Tukey double arcsine transformation was used to stabilize variances from extreme proportional estimates. *Any DBM refers to the pooling of all typologies within the specific level (individual, household/pair) and population (Children 0-18, Adults > 18, adult-child pair); for the typologies related to child with overnutrition + adult with undernutrition, any DBM could not be estimated as only one typology (child with overweight + adult with underweight) was available. ⁺ Mixed undernutrition indicates a combination of types of undernutrition.

References

1 The World Bank. World regions according to the World Bank. https://ourworldindata.org/grapher/world-regions-according-to-the-world-bank (accessed Aug 7, 2023).

2 The World Bank. The World by Income and Region. 2021. https://datatopics.worldbank.org/world-development-indicators/the-world-by-income-and-region.html (accessed Aug 8, 2023).

3 Barquera S, Peterson KE, Must A, *et al.* Coexistence of maternal central adiposity and child stunting in Mexico. *Int J Obes* 2007; **31**: 601–7.

4 Barreto SM, Passos VM, Lima-Costa MF. Obesity and underweight among Brazilian elderly: the Bambuí Health and Aging Study. *Cad saúde pública / Ministério da Saúde, Fundação Oswaldo Cruz, Esc Nac Saúde Pública* 2003; **19**: 605–12.

5 Bassete MN, Romaguera D, Gimenez MA, Lobo MO, Samman NC. Prevalence and determinants of the dual burden of malnutrition at the household level in Puna and Quebrada of Humahuaca, Jujuy, Argentina. *Nutr Hosp* 2014; **29**: 322–30.

6 Bernabé-Ortiz A, Quinteros-Reyes C, Carrillo-Larco RM. Double burden of malnutrition as a risk factor for overweight and obesity. *Rev Saude Publica* 2022; **56**: 93.

7 Caleyachetty R, Thomas GN, Kengne AP, *et al.* The double burden of malnutrition among adolescents: Analysis of data from the Global School-Based Student Health and Health Behavior in School-Aged Children surveys in 57 low- and middle-income countries. *Am J Clin Nutr* 2018; **108**: 414–24.

8 Conde WL, Monteiro CA. Nutrition transition and double burden of undernutrition and excess of weight in Brazil. *Am J Clin Nutr* 2014; **100**: 1617S-1622S.

9 Costa MH, de Souza AI, Braga MC, Batista M. Coexistence of anemia and vitamin A deficiency in women of childbearing age in the Northeast region of Brazil. *Rev Nutr J Nutr* 2013; **26**: 509–16.

10 De Menezes Toledo Florêncio TM, Da Silva Ferreiraa H, De França APT, Cavalcante JC, Sawaya AL. Obesity and undernutrition in a very-low-income population in the city of Maceió, northeastern Brazil. *Br J Nutr* 2001; **86**: 277–83.

11 Dieffenbach S, Stein AD. Stunted child/overweight mother pairs represent a statistical artifact, not a distinct entity. *J Nutr* 2012; **142**: 771–3.

12 Doak CM, Adair LS, Bentley M, Monteiro C, Popkin BM. The dual burden household and the nutrition transition paradox. *Int J Obes* 2005; **29**: 129–36.

13 Doak CM, Campos Ponce M, Vossenaar M, Solomons NW. The stunted child with an overweight mother as a growing public health concern in resource-poor environments: A case study from Guatemala. *Ann Hum Biol* 2016; **43**: 122–30.

14 Eckhardt CL, Torheim LE, Monterrubio E, Barquera S, Ruel MT. The overlap of overweight and anemia among women in three countries undergoing the nutrition transition. *Eur J Clin Nutr* 2008; **62**: 238–46.

15 Felix-Beltran L, Macinko J, Kuhn R. Maternal height and double-burden of malnutrition households in Mexico: stunted children with overweight or obese mothers. *Public Health Nutr* 2020; **24**: 106–16.

16 Ferreira HDS, Luna AA, Florêncio TMMT, Assunção ML, Horta BL. Short Stature Is Associated With Overweight but Not With High Energy Intake in Low-Income Quilombola Women. *Food Nutr Bull* 2017; **38**: 216–25.

17 Fooken J, Vo LK. Are stunted child – overweight mother pairs a real defined entity or a statistical artifact? *Econ Hum Biol* 2022; **47**. DOI:10.1016/j.ehb.2022.101199.

18 Freire WB, Silva-Jaramillo KM, Ramirez-Luzuriaga MJ, Belmont P, Waters WF. The double burden of undernutrition and excess body weight in Ecuador. *Am J Clin Nutr* 2014; **100**: 1636S-1643S.

19 Garrett J, Ruel MT. The coexistence of child undernutrition and maternal overweight: prevalence, hypotheses, and programme and policy implications. *Matern Child Nutr* 2005; **1**: 185–96.

20 Géa-Horta T, Silva RDCR, Fiaccone RL, Barreto ML, Velásquez-Meléndez G. Factors associated with nutritional outcomes in the mother-child dyad: A population-based cross-sectional study. *Public Health Nutr* 2016; **19**: 2725–33.

21 Ghattas H, Acharya Y, Jamaluddine Z, Assi M, El Asmar K, Jones AD. Child-level double burden of malnutrition in the MENA and LAC regions: Prevalence and social determinants. *Matern Child Nutr* 2020; **16**. DOI:10.1111/mcn.12923.

22 Gubert MB, Spaniol AM, Segall-Correa AM, Perez-Escamilla R. Understanding the double burden of malnutrition in food insecure households in Brazil. *Matern CHILD Nutr* 2017; **13**. DOI:10.1111/mcn.12347.

23 Jardim-Botelho A, Queiroz Gurgel R, Simeone Henriques G, *et al.* Micronutrient deficiencies in normal and overweight infants in a low socio-economic population in north-east Brazil. *Paediatr Int Child Health* 2016; **36**: 198–202.

24 Jones AD, Hoey L, Blesh J, Janda K, Llanque R, Aguilar AM. Peri-Urban, but Not Urban, Residence in Bolivia Is Associated with Higher Odds of Co-Occurrence of Overweight and Anemia among Young Children, and of Households with an Overweight Woman and Stunted Child. *J Nutr* 2018; **148**: 632–42.

25 Jones AD, Mundo-Rosas V, Cantoral A, Levy TS. Household food insecurity in Mexico is associated with the co-occurrence of

overweight and anemia among women of reproductive age, but not female adolescents. *Matern Child Nutr* 2017; **13**. DOI:10.1111/mcn.12396.

26 Jones-Smith JC, Fernald LCH, Neufeld LM. Birth Size and Accelerated Growth during Infancy Are Associated with Increased Odds of Childhood Overweight in Mexican Children. *J Am Diet Assoc* 2007; **107**: 2061–9.

27 Kroker-Lobos MF, Pedroza-Tobías A, Pedraza LS, Rivera JA. The double burden of undernutrition and excess body weight in Mexico. *Am J Clin Nutr* 2014; **100**: 1652S-8S.

28 Lee J, Houser RF, Must A, De Fulladolsa PP, Bermudez OI. Disentangling nutritional factors and household characteristics related to child stunting and maternal overweight in Guatemala. *Econ Hum Biol* 2010; **8**: 188–96.

29 Lee J, Houser RF, Must A, de Fulladolsa PP, Bermudez OI. Socioeconomic disparities and the familial coexistence of child stunting and maternal overweight in guatemala. *Econ Hum Biol* 2012; **10**: 232–41.

30 Lee J, Houser R, Must A, Palma P, Bermudez O. Association of the Familial Coexistence of Child Stunting and Maternal Overweight with Indigenous Women in Guatemala. *Matern Child Health J* 2017; **21**: 2102–13.

31 Lee GO, Gutierrez C, Castro Morillo N, Cevallos W, Jones AD, Eisenberg JNS. Multiple burdens of malnutrition and relative remoteness in rural Ecuadorian communities. *Public Health Nutr* 2021; **24**: 4591–602.

32 Lerm BR, Crochemore-Silva I, Costa JC, Victora CG. The double burden of malnutrition in under-five children at national and individual levels: observed and expected prevalence in ninety-three low- and middle-income countries. *Public Health Nutr* 2021; **24**: 2944–51.

33 Leroy JL, Habicht J-P, González de Cossío T, Ruel MT. Maternal education mitigates the negative effects of higher income on the double burden of child stunting and maternal overweight in rural Mexico. *J Nutr* 2014; **144**: 765–70.

34 Lourenço BH, Villamor E, Augusto RA, Cardoso MA. Influence of early life factors on body mass index trajectory during childhood: A population-based longitudinal analysis in the Western Brazilian Amazon. *Matern Child Nutr* 2015; **11**: 240–52.

35 Mendoza-Quispe D, Hernandez-Vasquez A, Miranda JJ, *et al.* Urbanization in Peru is inversely associated with double burden of malnutrition: Pooled analysis of 92,841 mother-child pairs. *OBESITY* 2021; **29**: 1363–74.

36 Oliveira MDN, Martorell R, Nguyen P, *et al.* Risk factors associated with hemoglobin levels and nutritional status among Brazilian children attending daycare centers in Sao Paulo city, Brazil Referencias. *Arch Latinoam Nutr* 2023; : 1–9.

37 Otten HS, Seferidi P. Prevalence and socioeconomic determinants of the double burden of malnutrition in mother-child pairs in Latin America and the Caribbean. *BMJ Nutr Prev Heal* 2022; **5**: 263–70.

38 Oviedo-Solis CI, Monterrubio-Flores EA, Cediel G, Denova-Gutierrez E, Barquera S. Trend of Ultraprocessed Product Intake Is Associated with the Double Burden of Malnutrition in Mexican Children and Adolescents. *Nutrients* 2022; **14**. DOI:10.3390/nu14204347.

39 Pajuelo Ramírez J, Miranda Cuadros M. La coexistencia de problemas nutricionales en niños menores de 5 años en el Perú 2007-2010. *An la Fac Med* 2016; **77**: 345.

40 Palma Gutierrez EJ. Prevalencia de la coexistencia de anemia y sobrepeso u obesidad en niños de 6 a 59 meses de edad y factores sociodemográficos asociados en el Perú. 2019.

41 Parra DC, Iannotti L, Gomez LF, *et al.* The nutrition transition in Colombia over a decade: A novel household classification system of anthropometric measures. *Arch Public Heal* 2015; **73**. DOI:10.1186/s13690-014-0057-5.

42 Parra DC, Gomez LF, Iannotti L, Haire-Joshu D, Sebert Kuhlmann AK, Brownson RC. Multilevel correlates of household anthropometric typologies in Colombian mothers and their infants. *Glob Heal Epidemiol Genomics* 2018; **3**. DOI:10.1017/gheg.2018.4.

43 Parra DC, Gomez LF, Iannotti L, Haire-Joshu D, Sebert Kuhlmann AK, Brownson RC. Maternal and familial correlates of anthropometric typologies in the nutrition transition of Colombia, 2000-2010. *Public Health Nutr* 2018; **21**: 2584–94.

44 Pomati M, Mendoza-Quispe D, Anza-Ramirez C, *et al.* Trends and patterns of the double burden of malnutrition (DBM) in Peru: a pooled analysis of 129,159 mother-child dyads. *Int J Obes* 2021; **45**: 609–18.

45 Popkin BM, Corvalan C, Grummer-Strawn LM. Dynamics of the double burden of malnutrition and the changing nutrition reality. *Lancet* 2020; **395**: 65–74.

46 Ramirez-Zea M, Kroker-Lobos MF, Close-Fernandez R, Kanter R. The double burden of malnutrition in indigenous and nonindigenous Guatemalan populations 1-4. *Am J Clin Nutr* 2014; **100**: 1644–51.

47 Raphaël D, Delisle H, Vilgrain C. Households with undernourished children and overweight mothers: Is this a concern for Haiti? *Ecol Food Nutr* 2005; **44**: 147–65.

48 Ribeiro-Silva RC, Silva NJ, Felisbino-Mendes MS, *et al.* Time trends and social inequalities in child malnutrition: nationwide estimates from Brazil's Food and Nutrition Surveillance System, 2009-2017. *Public Health Nutr* 2021; : 1–31.

49 Rivas PC, Gotthelf SJ. Anemia y estado nutricional en la población de la ciudad de salta anemia and nutritional status in population of Salta City. 2018 NS -.

50 Rivas-Marino G, Negin J, Salinas-Rodríguez A, *et al.* Prevalence of overweight and obesity in older Mexican adults and its

association with physical activity and related factors: An analysis of the study on global ageing and adult health. *Am J Hum Biol* 2015; **27**: 326–33.

51 Rodríguez Ramos F, Aradillas-García C, Díaz-Barriga F, Padrón Salas A. Intake of macronutrients and micronutrients in adolescents of an indigenous community in San Luis Potosí, Mexico . *Rev Esp Nutr Comunitaria* 2013; **19**: 152–8.

52 Rodríguez-Zúñiga MJ. Obesidad, sobrepeso y anemia en niños de una zona rural de Lima, Perú. *Med (Buenos Aires)* 2015; **75**: 379–83.

53 Samper-Ternent R, Michaels-Obregon A, Wong R. Coexistence of Obesity and Anemia in Older Mexican Adults. *Ageing Int* 2012; **37**: 104–17.

54 Sanson-Rosas AM, Bernal-Rivas J, Kubow S, Suarez-Molina A, Melgar-Quinonez H. Food insecurity and the double burden of malnutrition in Colombian rural households. *Public Health Nutr* 2021; **24**: 4417–29.

55 Sarmiento OL, Parra DC, Gonzalez SA, Gonzalez-Casanova I, Forero AY, Garcia J. The dual burden of malnutrition in Colombia. *Am J Clin Nutr* 2014; **100**: 1628S-1635S.

56 Sawaya AL, Martins PA, Grillo LP, Florêncio TT. Long-term effects of early malnutrition on body weight regulation. *Nutr Rev* 2004; **62**: S127-33.

57 Severi C, Moratorio X. Double burden of undernutrition and obesity in Uruguay. *Am J Clin Nutr* 2014; **100**: 1659S-1662S.

58 Syed S, Addo OY, De La Cruz-Góngora V, Ashour FAS, Ziegler TR, Suchdev PS. Determinants of anemia among school-aged children in Mexico, the United States and Colombia. *Nutrients* 2016; **8**: 1–15.

59 Temponi HR, Velasquez-Melendez G. Prevalence of double burden on malnutrition at household level in four latin america countries. *Rev Bras Saude Matern Infant* 2020; **20**: 27–35.

60 Uzêda JCO, Ribeiro-Silva RDC, Silva NDJ, *et al.* Factors associated with the double burden of malnutrition among adolescents, National Adolescent School-Based Health Survey (PENSE 2009 and 2015). *PLoS One* 2019; **14**. DOI:10.1371/journal.pone.0218566.

61 Varela-Silva MI, Dickinson F, Wilson H, Azcorra H, Griffiths PL, Bogin B. The nutritional dual-burden in developing countries--how is it assessed and what are the health implications? *Coll Antropol* 2012; **36**: 39–45.

62 Williams AM, Guo J, Yaw Addo O, *et al.* Intraindividual double burden of overweight or obesity and micronutrient deficiencies or anemia among women of reproductive age in 17 population-based surveys. *Am J Clin Nutr* 2020; **112**: 468S-477S.
